# KLF14 regulates the growth of hepatocellular carcinoma cells via its modulation of iron homeostasis through the repression of iron-responsive element-binding protein 2

**DOI:** 10.1186/s13046-022-02562-4

**Published:** 2023-01-05

**Authors:** Hui Zhou, Junru Chen, Mingjie Fan, Huajian Cai, Yufei Dong, Yue Qiu, Qianqian Zhuang, Zhaoying Lei, Mengyao Li, Xue Ding, Peng Yan, Aifu Lin, Shusen Zheng, Qingfeng Yan

**Affiliations:** 1 College of Life Science, Zhejiang University, Hangzhou, 310058 Zhejiang China; 2 Division of Hepatobiliary and Pancreatic Surgery, Department of Surgery, The First Affiliated Hospital, Zhejiang University School of Medicine, Hangzhou, 310003 Zhejiang China; 3 Department of Pediatrics, The First Affiliated Hospital, School of Medicine Zhejiang University, Hangzhou, 310003 Zhejiang China; 4 Key Laboratory for Cell and Gene Engineering of Zhejiang Province, Hangzhou, 310058 Zhejiang China

**Keywords:** KLF14, IRP2, Iron metabolism, Fluphenazine, Hepatocellular carcinoma

## Abstract

**Background:**

Hepatocellular carcinoma (HCC) is a multifactor-driven malignant tumor with rapid progression, which causes the difficulty to substantially improve the prognosis of HCC. Limited understanding of the mechanisms in HCC impedes the development of efficacious therapies. Despite Krüpple-Like factors (KLFs) were reported to be participated in HCC pathogenesis, the function of KLF14 in HCC remains largely unexplored.

**Methods:**

We generated KLF14 overexpressed and silenced liver cancer cells, and nude mouse xenograft models for the in vitro and in vivo study. Luciferase reporter assay, ChIP-qPCR, Co-IP, immunofluorescence were performed for mechanism research. The expression of KLF14 in HCC samples was analyzed by quantitative RT-PCR, Western blotting, and immunohistochemistry (IHC) analysis.

**Results:**

KLF14 was significantly downregulated in human HCC tissues, which was highly correlated with poor prognosis. Inhibition of KLF14 promoted liver cancer cells proliferation and overexpression of KLF14 suppressed cells growth. KLF14 exerts its anti-tumor function by inhibiting Iron-responsive element-binding protein 2 (IRP2), which then causes transferrin receptor-1(TfR1) downregulation and ferritin upregulation on the basis of IRP-IREs system. This then leading to cellular iron deficiency and HCC cells growth suppression in vitro and in vivo. Interestingly, KLF14 suppressed the transcription of IRP2 via recruiting SIRT1 to reduce the histone acetylation of the IRP2 promoter, resulting in iron depletion and cell growth suppression. More important, we found fluphenazine is an activator of KLF14, inhibiting HCC cells growth through inducing iron deficiency.

**Conclusion:**

KLF14 acts as a tumor suppressor which inhibits the proliferation of HCC cells by modulating cellular iron metabolism via the repression of IRP2. We identified Fluphenazine, as an activator of KLF14, could be a potential compound for HCC therapy. Our findings therefore provide an innovative insight into the pathogenesis of HCC and a promising therapeutic target.

**Supplementary Information:**

The online version contains supplementary material available at 10.1186/s13046-022-02562-4.

## Introdution

Hepatocellular carcinoma (HCC) is the major type of liver cancer with the characteristic of rapid progression and postoperative recurrence, which represents 80–90% of primary liver cancers [1, 2]. The number of HCC cases is rising annually [3]. Despite with the improvement of surgical technology and drug treatment, it is still difficult to substantially improve the prognosis of HCC [4]. It is urgent to improve the understanding of the mechanisms of HCC and identify promising targets to open up the opportunity for novel therapeutic interventions.

Krüpple-Like factor 14 (KLF14), also known as BTEB5, is a member of KLF family. KLFs show conserved C2H2-type zinc finger domains at their C-termini and acts to regulate biological processes via their binding to the GC-rich sites in the regulatory regions of their target genes [5]. Accumulated experimental data has demonstrated that KLFs are involved in pathogenesis of HCC [6]. Recently, KLF14 attracted attention for its role in multiple biological processes related to metabolism and immunity [7–10]. Importantly, KLF14 showed a lower expression in breast cancer and colorectal cancer indicating a tumor suppression effect [11–14]. However, no evidence illustrates the function of KLF14 in HCC. In light of this, the molecular mechanism of KLF14 in HCC remains open for study.

Iron homeostasis is critical for cell proliferation and growth [15]. Iron deficiency impairs cell proliferation and growth, and an overload of cellular iron catalyzes the generation of free radicals [16]. Iron regulatory proteins (IRPs) comprised of iron-regulatory protein 1 and 2 (IRP1 and IRP2) are the key regulators of the iron responsive elements (IREs). IRPs bind to target elements in the mRNA 5’untranslated region (5’UTR) to suppress translation such as ferritin (FH) and ferroportin (FPN), or the 3’UTR to prevent mRNA degradation such as transferrin receptor-1 (TfR1) and divalent metal transporter 1 (DMT1) [17]. Numerous work elucidated that cancer often occurs with an altered iron metabolism. As liver is the major site of iron storage, which makes liver the most sensitive organ in cases of iron overloading [18]. Clinical and animal studies suggested that iron overloading is a key reason for liver cancer [19, 20]. Correspondingly, inducing cellular iron depletion is a promising approach for HCC therapy [21–23]. IRPs were identified as major regulators of cellular iron metabolism, suggesting molecules that target IRPs as potential therapeutic targets for HCC. Since the mechanism of IRPs is poorly understood, it is worth further investigation in HCC.

Here, we find that KLF14 is significantly downregulated in HCC patients and inhibits the proliferation of HCC cells by modulating cellular iron metabolism via the repression of IRP2. The inhibition effect of KLF14 on HCC cells was dependent on iron, which suggested a novel function of KLF14 in regulating cellular iron metabolism and cell growth. Noteworthily, we identified a KLF14 activator, fluphenazine, which markedly reduces the cellular labile iron pool (LIP) content and suppresses the growth of HCC cells in vitro and in vivo. Collectively, this study demonstrates an unrecognized mechanism of KLF14 mediating cellular iron metabolism and suppressing the proliferation of HCC. KLF14 could serve as a promising therapeutic target for the treatment of HCC, suggesting a potential translational value in the therapy of HCC.

## Methods and materials

### Tumor samples

In this study, 69 fresh specimens of HCC tissues were collected to detect the mRNA and protein levels of KLF14 and IRP2 by qRT-PCR and Western blot. Samples were collected from the First Affiliated Hospital, Zhejiang University School of Medicine, Zhejiang, China. The study was approved by the Ethical Review Committee of this hospital. Written informed consent was obtained in compliance with the guidelines of the Declaration of Helsinki. Human HCC tissue chip containing both tumor and the adjacent tissue sections (TFHCC-01) was purchased from Shanghai TuFei Biotech. Antibodies against KLF14, IRP2 and TfR1 used for IHC staining were from Abcam (ab244475), Proteintech (23829–1-AP) and ABclonal (A5865) respectively.

### Cell culture and chemicals

293T cells were purchased from ATCC. HepG_2_ cells were obtained from Prof Aifu Lin (Zhejiang University, China) and Huh7 cells were obtained from Prof Xiaoyuan Lian (Zhejiang University, China). MIHA, Hep3B, HCC-LM3, PLC/PRF5, HLE and Sun387 cells were obtained from Prof Shusen Zheng (Zhejiang University, China). 293T, MIHA, HepG_2_, Hep3B, HCC-LM3, PLC/PRF5, HLE cells were cultured in DMEM (Gibco, Grand Island, NY, USA) with 10% fetal bovine serum (Gibco). Huh7 cells were maintained in DMEM (Gibco) with 10% fetal bovine serum and 1% GlutMAX (Gibco). Sun387 cells were cultured in RPMI 1640 (Gibco, Grand Island, NY, USA) with 10% fetal bovine serum (Gibco). Trichostatin A (TSA, MCE, New Jersey, USA), Nicotinamide (NAM, MCE) were dissolved in dimethyl sulfoxide (DMSO). Fluphenazine hydrochloride (FPZ, National Institutes for Food and Drug Control, Beijing, China), Ferric ammonium citrate (FAC, Sigma-Aldrich, St. Louis, MO) and Deferoxamine mesylate (DFO, MCE) were dissolved in phosphate buffer saline (PBS). Iron dextran (National Institutes for Food and Drug Control) was dissolved in 0.9% NaCl.

### Plasmids and transfection

KLF14-WT-3 × Flag, KLF14-ΔZF3–3 × Flag, KLF14-M4–3 × Flag and the IRP2 coding sequence were cloned into pCDH-CMV-Puro vector. The pLKO.1-shRNAs targeting KLF14 nucleotides were 5′-CCGGGCTGCACCAAAGCCTATTACACTCGAGTGTAATAGGCTTTGGTGCAGCTTTTTG-3′ (sh-KLF14–1), 5′-CCGGTCATCCAGATATGATCGAGTACTCGAGTACTCGATCATATCTGGATGATTTTTG-3′ (sh-KLF14-2). These plasmids were co-transfected with the plasmid psPAX2 and pMD2.G into 293 T cells, and the viruses were harvested at 48 h and 72 h after transfection to transduce HepG_2_ and Huh7 cells, followed by selection with 2 μg/mL puromycin. The stable cell lines with KLF14 overexpression or KLF14 knockdown were screened by qRT–PCR and western blot assay and used for following functional studies. The pLKO.1-shRNAs targeting IRP2, SIRT1 and SIRT5 nucleotides were 5′- CCGGGGACCTAAATCAGAATCATAGCTCGAGCTATGATTCTGATTTAGGTCCTTTTTG-3′(sh-IRP2–1),5′-CCGGGCAAACATGTGTCCGGAATATCTCGAGATATTCCGGACACATGTTTGCTTTTTG-3′(sh-IRP2–2),5′-CCGGGCAAAGCCTTTCTGAATCTATCTCGAGATAGATTCAGAAAGGCTTTGCTTTTTG-3′(sh-SIRT1–1),5′-CCGGGATGATCAAGAGGCAATTAATCTCGAGATTAATTGCCTCTTGATCATCTTTTTG-3′(sh-SIRT1–2),5′-CCGGGCTACGAACAGATTCAGGTTTCTCGAGAAACCTGAATCTGTTCGTAGCTTTTTG-3′(sh-SIRT5).

Plasmids transfection were carried out using Lipofectamine 2000 (11,668,019, Invitrogen, Carlsbad, CA, USA) according to the manufacturer’s instructions.

### Quantitative real-time PCR assay

Total cellular RNA was extracted using Trizol Reagent (Invitrogen; Thermo Fischer Scientific, Inc., Waltham, MA) and reverse transcribed into complementary DNA using the PrimeScript RT Reagent kit with genomic DNA Eraser (TaKaRa, Japan). The detection the relative expression of target genes was conducted using a Bio-Rad CFX96 quantitative real-time PCR system with the SYBR-Green method (TaKaRa, Japan). PCR was conducted at 95 °C for 2 min, followed by 40 cycles of 95 °C for 10 sec, 58 °C for 30 sec, and 72 °C for 30 sec. GAPDH served as internal controls. The expression of relative target genes was analyzed using the 2^-∆∆Cq^ method. The sequences of relative human primer pairs are listed in Supplementary Table 1.

### Western blot

Western blot analysis was performed as previously described [24]. Antibodies in this work were anti-KLF14 (ab85476; Abcam, Cambridge, MA, USA), anti-IRP1 (12406–1-AP, Proteintech, 1:1000), anti-IRP2 (23829–1-AP, Proteintech, 1:1000), anti-TfR1 (A5865, ABclonal, 1:1000), anti-Ferritin H (A19544, ABclonal, 1:1000), anti-SIRT1 (A17307, ABclonal, 1:1000), anti-GAPDH (M20006, Abmart, 1:5000), anti-Flag (M20008, Abmart, 1:5000, 1:1000), anti-HA (ab1424，abcam, 1:1000), anti-Actin (ab8227, abcam, 1:1000).

### Luciferase reporter assay

To construct the human *IRP2* promoter reporter (PGL4-*IRP2*-luc), the product of 1 kb upstream/downstream of the translation start site of the human *IRP2* gene (− 1000 to + 1000) was amplified from human genomic DNA, and then ligated into the pGL4-luciferase reporter vector (Promega) to generate a PGL4-*IRP2*-luc plasmid. HepG_2_ cells were seeded into 12-wells and transfected with the PGL4-*IRP2*-luc plasmid and the above indicated expression plasmids using Lipofectamine 2000 (Life Technologies). Cells were then cultured for 48 hours beyond transfection, then measured luciferase activities using a Dual Luciferase Reporter Assay System Kit (Promega).

To prepare the human *KLF14* promoter–driven luciferase reporter, the amplified product 1 kb upstream/downstream of the translation start site of the human *KLF14* gene (− 1000 to + 1000) was ligated into the pGL4-luciferase reporter vector to generate a PGL4-*KLF14*-luc plasmid. To identify the activation of fluphenazine hydrochloride on *KLF14*, HepG_2_ cells were transfected with the PGL4-*KLF14*-luc plasmid for 6 hours and then this compound was added into medium with fluphenazine treatment for 48 hours.

### ChIP-qPCR assay

A chromatin immunoprecipitation (ChIP) assay was performed following the published protocol from Varun Sasidharan Nair [25]. All sample cells were treated with 1% formaldehyde. Then use the nuclei lysis buffer to treat the crosslinked samples to obtain the nuclei. The nuclei lysis buffer was sonicated into chromatin fragments of 200-500 bp using a Bioruptor. The fragmented chromatin was immunoprecipitated using antibodies for 4 h, and then captured using protein A-Sepharose beads. Chromatin was eluted from the beads and treated with proteinase K which was then followed by phenol/chloroform extraction and ethanol precipitation to obtain purified DNA. The DNA samples were used as the template for ChIP-qPCR， the primers are listed in Supplementary Table 2. The Antibodies for this work were anti-H3K9ac (A7255, ABclonal), anti-H4K16ac (A5280, ABclonal), anti-IgG (#2729, CST).

### Co-IP assay

Cells were collected and washed with cold PBS, then lysed with cold lysis buffer (50 mmol/L Tris-HCl (pH 7.4), 150 mmol/L NaCl, 1 mmol/L EDTA, 1 mmol/L DTT, and 0.5% NP-40) containing protease inhibitor cocktail (Roche) on ice for 30 min. Immunoprecipitation was implemented by incubating lysate with Flag beads (Sigma) at 4 °C overnight. After incubation, the beads were washed, resuspended in 30 μl of 2× loading buffer and boiled for 10 min. Proteins were then separated by using SDS–PAGE (10% SDS) and transferred to a nitrocellulose membrane for immunoblot detection.

### Immunofluorescence

Cells were seeded into 24-wells and grown on chamber slides. Cells were then transfected with KLF14-EGFP、SIRT1-RFP or SIRT5-RFP for 48 hours. Cells were washed in PBS for twice and fixed with 4% paraformaldehyde (Sangon Biotech, Shanghai, China) for 15 min at room temperature. They were then washed with PBS for another two times, and then stained with 1 μg/mL DAPI for 10 min at room temperature. Fluorescence intensities were detected and measured using a Zeiss LSM710 laser scanning confocal microscope (Nikon) and the Zen2011 analysis software.

### Molecular simulation and docking analysis

The initial coordinates and structure model of three zinc finger domains of KLF14 were predicted using SWISS-MODEL algorithm and the homologous template models of KLF14 was acquired from the protein data bank (PDB entry 2WBS.A) [26–30]. The coordinates and structure model of SIRT1 were acquired from the protein data bank (PDB entry 5BTR). Coordinates were prepared using PyMol 2.3.0 with all ligand and water molecules removed and hydrogen atoms added. Molecular docking models of KLF14-SIRT1 were predicated by using Z-DOCK 3.0.2 and GrammX prediction algorithms [31, 32]. Docking poses were evaluated and sorted using MolDock Score, the top scoring pose was selected as the final conformation. The intermolecular force and the protein-protein interaction interfaces of all docking complex models were detected and performed using PyMol 2.3.0.

### Measurement of intracellular iron level

In viable cells, Calcein-AM (calcein-acetoxymethyl) enters and becomes fluorescent upon hydrolysis via esterase enzyme. Its fluorescence is quenched by intracellular iron content [33, 34]. Cells were incubated with 100 nM Calcein-AM (BioLegend) for 30 min at 37 °C in PBS. After Calcein-AM loading, the cells were trypsinized, washed and, re-suspended in PBS without Calcein-AM. They were then plated in 96-cell plates. Fluorescence was monitored using multifunctional enzyme mark at λex 488 nm and λem 518 nm (Thermo, USA) where a low Calcein-AM fluorescence intensity indicates high intracellular free iron level.

### Cell growth assay and colony formation assay

For the cell growth assay, HepG_2_ and Huh7 stable cell lines were seeded into 12-well plates. With drug incubation, fluphenazine hydrochloride, FAC or DFO were added into medium after cells were planted for 12 hours. Cells were cultured for 4 days and were counted every 24 hours.

For HepG_2_ and Huh7 cells, stably overexpressed or knockdown cell were seeded into 6-well plates at a density of 600 cells per well. The cells were maintained for 10–12 days until the clones became visible. The colonies were washed with PBS for twice and fixed with 4% paraformaldehyde (Sangon Biotech, Shanghai, China) at room temperature for 15 min, again washed with PBS for twice, and then stained with 1% Crystal Violet (Sigma) for 10 min at room temperature.

### Cell apoptosis analysis

For the cell apoptosis assay, an Annexin V-FITC apoptosis assay kit (Absin Bioscience Inc., Shanghai, China) was used according to the manufacturer’s protocol. Beyond collection, cells were washed with cold PBS, and then cells were suspended in Annexin-V binding buffer. An incubation with FITC at room temperature for 10 min, cells were dyed with propidium iodide (PI) at room temperature for 5 min. The cell apoptosis was evaluated using a flow cytometer.

### The in vivo tumorigenesis assay

All animal studies in this study were approved by the committee on Use and Care of Animals at the Zhejiang University (No. ZJU20210201), and conducted in accordance with their guidelines. Four-week-old BALB/c nude mice were purchased from GemPharmatech (Nanjing, China) and randomly divided into groups (*n* = 6). HepG_2_ cells were injected into the back region of mice. Tumor size and body weight of the mice were measured every 3 days. Tumor volumes were calculated according to the following equation: volume = length (mm) × width (mm) ^2^ / 2. At the conclusion of the experiment, tumors were harvested and weighed.

### Immunohistochemistry (IHC)

Tumors were harvested and fixed with 4% paraformaldehyde, then embedded in paraffin. Embedded samples were deparaffinized and stained with H&E (Proteintech) and Perl’s Blue (Proteintech) routinely. Additionally, the embedded tissues were dewaxed and rehydrated. Antigen retrieval was then conducted. After incubation of primary and secondary antibodies, the slides were dehydrated and stabilized with mounting medium. The samples were observed and photographed using a Nikon ECLIPSE Ni-U microscope. Antibodies in this work were anti-Ki67 (27309–1-AP，Proteintech, 1:100), anti-cleaved caspase 3 (9661, Cell Signaling Technology, 1:100), anti-IRP2 (23829–1-AP, Proteintech, 1:100) and anti-KLF14 (ab244475, Abcam, 1:75).

### Statistical analysis

All experiments were independently repeated at least three times. The statistical significance of the data between two experimental groups was determined and analyzed using unpaired 2-sided Student’s t-test. The Kaplan–Meier survival analysis was conducted with log-rank test. Besides, correlational analysis of two genes was tested by Pearson rank correlation analysis. Categorical data were analyzed by Fisher’s exact test. Among all the data sets, *p* values less than 0.05 were considered significant. (denoted as *p < 0.05, **p < 0.01, ***p < 0.001).

## Results

### Down-regulation of KLF14 indicates a poor prognosis in HCC

**Fig. 1:**
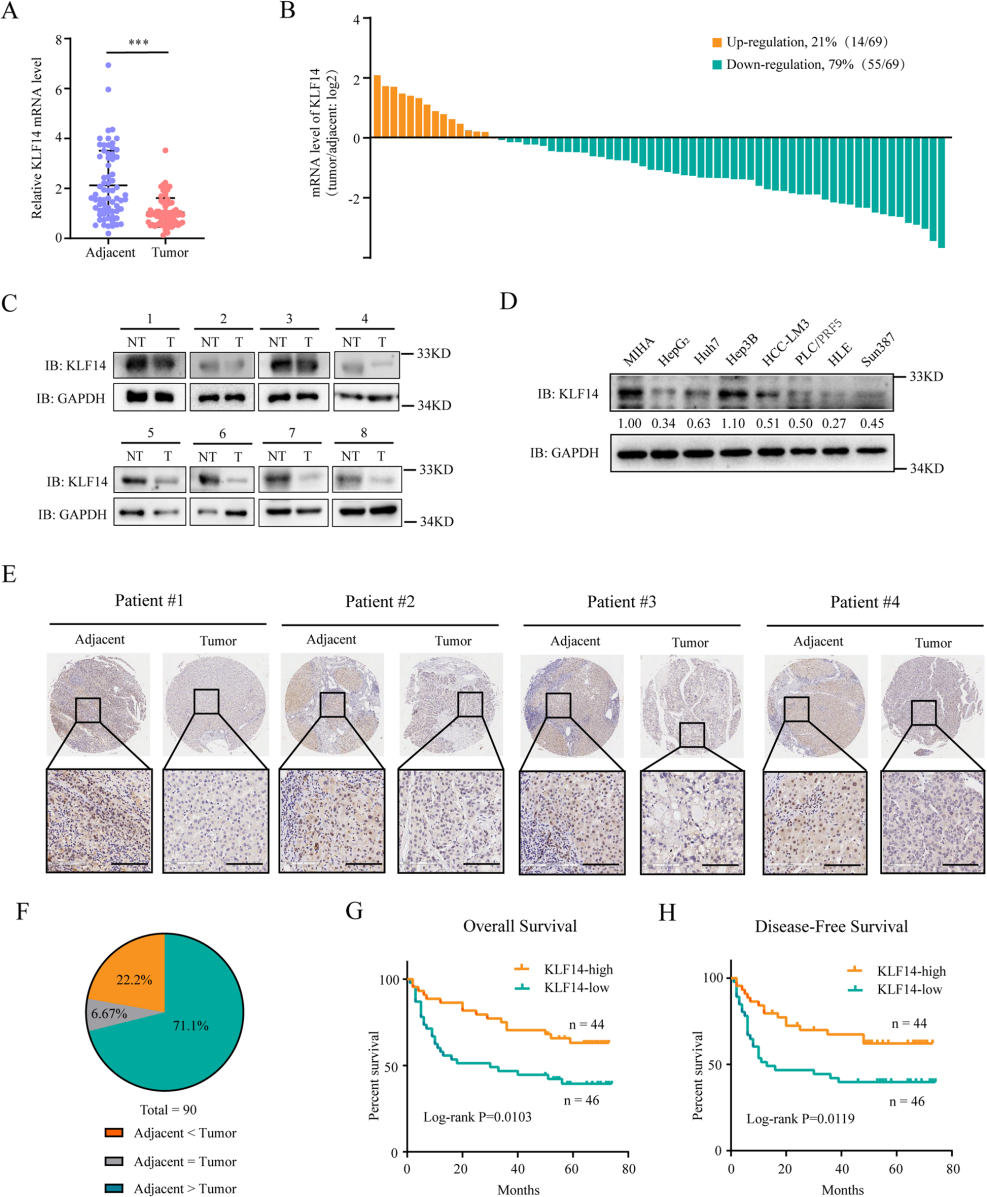
Downregulation of KLF14 is correlated with poor prognosis in HCC. **A** Relative mRNA level of KLF14 in 69 paired tumor tissues and adjacent tissues of HCC. **B** Expression ratios of “tumor tissues/adjacent tissues” in HCC were calculated and shown as histogram (*n* = 69). **C** The protein level of KLF14 in tumor tissues and its matched adjacent tissues. **D** The protein level of KLF14 in normal liver cells and HCC cell lines. **E** Representative Immunohistochemistry staining of KLF14 in HCC tumor and adjacent tissues (*n* = 90) were exhibited (scale bar, 100 μm). **F** Alteration of KLF14 expression in paired samples are depicted in the pie chart. **G-H** Kaplan-Meier analyses were conducted to evaluate the overall survival **(G)** and disease-free survival **(H)** according to the expression of KLF14 in HCC tissue chip (*n* = 90). Data are presented as the mean ± SD. Two-tailed unpaired Student’s T-tests were performed. ***P < 0.001

To determine the role of KLF14 in HCC, we firstly analyzed the expression of KLF14 in HCC by qRT-PCR and Western blot. The results showed that the mRNA level of KLF14 was significantly down-regulated in HCC tumor tissues compared with adjacent tissues (Fig. 1A). There was approximately 79% of samples with lower mRNA level of KLF14 (Fig. 1B). The protein level of KLF14 in patients with HCC and HCC cell lines were analyzed subsequently. Results showed that KLF14 was downregulated in HCC tumor tissues and most HCC cell lines (Fig. 1C, D). IHC staining of KLF14 in HCC samples further confirmed that 71.1% of samples presented a significantly lower expression in tumor tissues than that in paired adjacent tissues (Fig. 1E, F). Besides, KLF14 expression was significantly correlated with tumor size, TNM stage, serum AFP level and portal vein tumor thrombus (PVTT) in patients with HCC (Supplementary Table 3). The Kaplan–Meier analysis revealed that a lower expression of KLF14 is correlated with poor prognosis in overall survival (*P* = 0.0103) and disease-free survival (*P* = 0.0119) (Fig. 1G, H). Together, these results showed that KLF14 is significantly downregulated in HCC and it is correlated with poor prognosis.

### KLF14 inhibits the proliferation of HCC cells and regulates cellular iron homeostasis

**Fig. 2:**
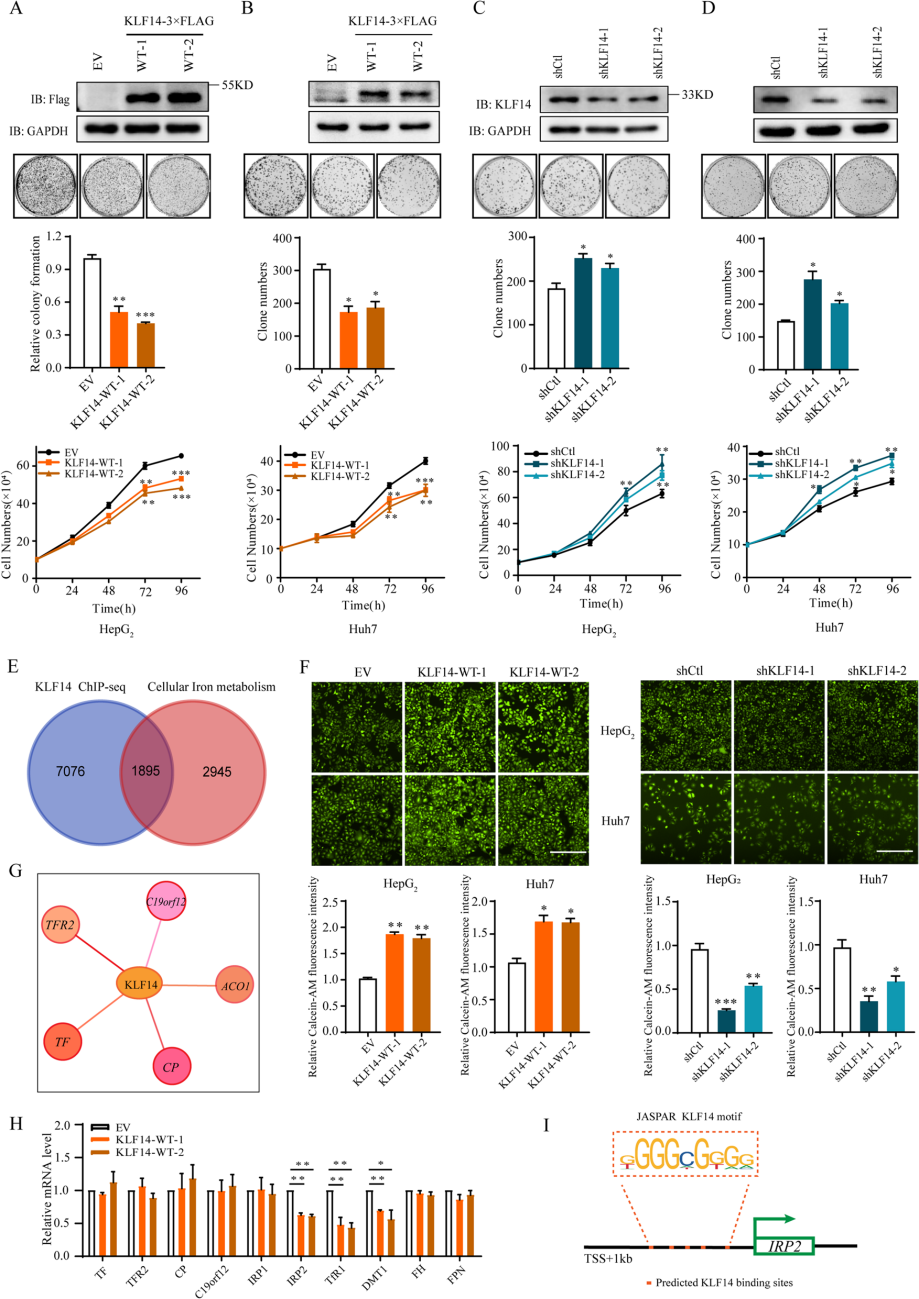
KLF14 inhibits the growth and proliferation of HCC cells and regulates cellular iron metabolism. **A, B** Overexpression of KLF14 in HepG_2_ and Huh7 cells inhibited cell growth and colony formation. Western blotting analysis of KLF14 expression in liver cancer cells. **C, D** KLF14 knockdown promoted cell growth and colony formation. **E** Venn diagram of ChIP-seq and cellular iron metabolism related genes showing that 1895 genes related to cellular iron metabolism were found to be potential target genes of KLF14. **F** Relative cellular LIP content in KLF14 overexpressed cells and KLF14 knockdown cells were measured using a calcein-AM assay at 24 h (*up* panel) and 36 h (*down* panel). **G** The five genes among 1895 genes, which are related to cellular iron metabolism (related score > 50), showed enrichment for KLF14 binding peaks in data of ChIP assay and the presence of the proposed KLF14 binding motif. **H** Relative mRNA levels of the five genes and the key genes involved in IRPs-IRE system. **I** Potential binding sites of KLF14 in IRP2 promoter. All the results are presented as means ± SD from three independent experiments. Two-tailed unpaired Student’s T-tests were performed. *P < 0.05, **P < 0.01 and ***P < 0.001

To determine the effects of KLF14 on liver cancer cells. HepG_2_ and Huh7 stable cell lines were constructed with KLF14 overexpression or knockdown (Supplementary Fig. 1A). Overexpressed KLF14 significantly inhibited cell growth and clonogenic survival (Fig. 2A, B). Conversely, cells with KLF14 knockdown grew faster and exhibited enhanced clonogenic formation as compared with control cells (Fig. 2C, D). These results provided the initial evidence that KLF14 plays a critical role in liver cancer cells growth.

KLF14 has been reported to modulate the expression of 385 trans-genes in adipose tissue [7]. Several genes related to cellular iron metabolism such as *ACO1* (*IRP1*) were included in the trans-genes, we thus combined the trans-genes regulated by KLF14 and cellular iron metabolism related genes from gene cards, 105 genes were found to be related to cellular iron metabolism, suggesting a potential role of KLF14 in the cellular iron metabolism (data not shown). Considering that iron is related to HCC, we analyzed the data obtained by chromatin immunoprecipitation followed by deep sequencing (ChIP-seq) of KLF14 overexpressed HEK293 T cells [35] (*P* = 2 × 10^− 4^) and cellular iron metabolism related genes from gene cards. 1895 genes were found to be potential target gens of KLF14 (Fig. 2E), which suggested that KLF14 is highly correlated with cellular iron metabolism. To identify whether such KLF14 involvement in the iron metabolism occurs, we then applied calcein-AM assay to investigate LIP concentration [36]. Results showed that the fluorescence was higher in KLF14 overexpressed cells, which indicated that cellular LIP concentration was significantly decreased. Correspondingly, the intracellular iron concentration increased in KLF14-knockdown cells (Fig. 2F). These results suggested that KLF14 plays a crucial role in cellular iron metabolism. *TF*, *TFR2*, *CP*, *ACO1* (*IRP1*) and *C19orf12* were the five genes among the 1895 genes, which are highly related to cellular iron metabolism (related score > 50), showed enrichment for KLF14 binding peaks in data of ChIP assay and the presence of the proposed KLF14 binding motif (Fig. 2G). The mRNA levels of the five genes and IRPs-IRE system related genes including *FH* and *FPN* changed minimally. Interestingly, we found that *IRP2*, *TfR1* and *DMT1* were strongly repressed by KLF14 (Fig. 2H). IRP2 shares high homology with IRP1 and is responsible for the mRNA degradation of TfR1 and DMT1. Moreover, there showed potential binding sites of *IRP2* promoter [37] for KLF14 (Fig. 2I). These findings indicated that KLF14 suppressed HCC cells growth and may regulate cellular iron metabolism through targeting IRP2.

### KLF14 represses the expression of IRP2 in zinc finger 3 dependent manner

**Fig. 3:**
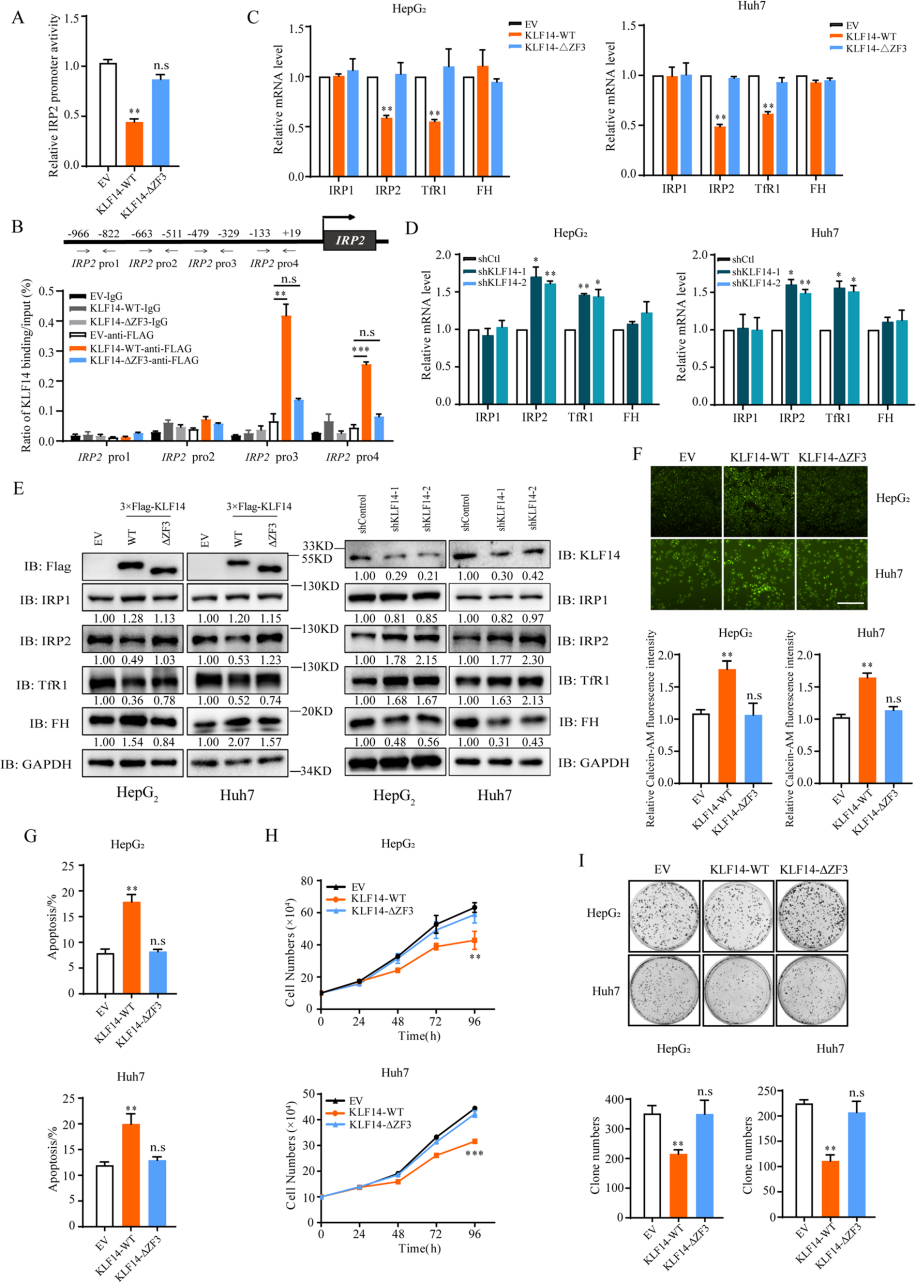
KLF14 suppresses IRP2 expression and its suppression is dependent on zinc finger 3. **A** Human IRP2 promoter luciferase reporter was transfected with KLF14-WT-3 × Flag or KLF14-ΔZF3–3 × Flag (deletion of zinc finger 3) in HepG_2_ cells for 48 h. Relative luciferase activity was then analyzed. **B** ChIP assay revealed significant enrichment of KLF14 protein on the IRP2 promoter in HepG_2_ cells. Schematic diagram of primer pairs of the human *IRP2* promoter region (*up* panel) in the ChIP assay and real-time PCR analysis. HepG_2_ cells transfected with KLF14-WT-3 × Flag or KLF14-ΔZF3–3 × Flag were harvested (with 3xFlag-Vector as control). Cross-linked samples were immunoprecipitated with anti-FLAG antibody, and the precipitated DNA fragments were subjected to qRT-PCR in the *IRP2* promoter regions. **C, D** Relative mRNA levels of IRP1, IRP2, TfR1 and FH (normalized with GAPDH mRNA) in KLF14-overexpressed (**C**) or KLF14-silenced (**D**) HepG_2_ and Huh7 cells were examined by qRT-PCR. **E** Western blotting was conducted to test the expression of IRP1, IRP2, TfR1, FH and GAPDH (control) in KLF14-WT-3 × Flag, KLF14-ΔZF3–3 × Flag overexpressed and KLF14-silenced HepG_2_ and Huh7 cells. **F** The relative cellular LIP contents in KLF14-WT-3 × Flag and KLF14-ΔZF3–3 × Flag overexpressed cells were measured at 24 h (*up* panel) and 36 h (*down* panel). **G** The cell apoptosis of KLF14-WT-3 × Flag and KLF14-ΔZF3–3 × Flag overexpressed cells were investigated by flow cytometry. **H, I** Cell growth curve (**H**) and Colony formation ability (**I**) of KLF14-WT-3 × Flag and KLF14-ΔZF3–3 × Flag overexpressed cells. All results are presented as means ± SD from three independent experiments. Two-tailed unpaired Student’s T-tests were performed. n.s, not significant, *P < 0.05, **P < 0.01 and ***P < 0.001

To further determine whether KLF14 regulates IRP2 transcription, we conducted a luciferase reporter assay through the PGL4-*IRP2*-luc system. Indeed, KLF14 showed a strong inhibitory effect on *IRP2*-promoter luciferase activity (Fig. 3A). Zinc finger domains were reported to be critical for KLF14 binding to GC-rich sites at the promoter of its target genes. Interestingly, zinc finger 3 (ΔZF3) deletion abolished the inhibition effects of KLF14 on IRP2 but not zinc finger 1 or 2 (Supplementary Fig. 2A). *IRP2*-promoter luciferase activity assay showed a similar result in KLF14-ΔZF3-transfected cells (Fig. 3A). Consistently, the ChIP assay result suggested that the zinc finger 3 was a necessary factor for KLF14 targeting in the region (− 479 bp – + 19 bp) of the *IRP2* promoter (Fig. 3B). To further understand the mechanism of IRP2 transcriptional regulation, we tested the mRNA and protein level of IRP2 and iron related genes in KLF14 overexpressed and silenced HCC cells. The results showed that the expression of IRP2 elevated upon KLF14 knockdown and downregulated upon KLF14 overexpressed (Fig. 3C, D). Concurrently, the mRNA and protein levels of TfR1 was downregulated with KLF14 overexpression and increased obviously with KLF14 silenced, and DMT1 shows the same trend (Fig. 3C-E and Supplementary Fig. 2B, C). The protein level of ferritin H (FH) was significantly upregulated with KLF14 overexpression and reduced with KLF14 knockdown, but its mRNA level remained unchanged. Moreover, the expression of IRP2 and iron related genes were not changed with zinc finger 3 (KLF14-ΔZF3) deletion, which indicated that KLF14-mediated down-regulation of IRP2 expression was dependent on the zinc finger 3. Collectively, these results suggested that KLF14 regulated the expression of IRP2 in a zinc finger 3 dependent manner.

Furthermore, the effect of KLF14 on cellular LIP concentration was diminished upon the deletion of zinc finger 3 (Fig. 3F). KLF14-overexpressed liver cancer cells displayed increased apoptosis rates, which was reversed with zinc finger 3 depleted (Fig. 3G and Supplementary Fig. 2D). Along with this, depleting zinc finger 3 of KLF14 markedly attenuated the suppression of full length KLF14 on cell growth and clonogenic formation (Fig. 3H, I). Together, these results suggested that KLF14 is a transcription repressor of IRP2, regulating cellular iron homeostasis, cell proliferation and survival.

### IRP2 is the functional target of KLF14 in iron-related HCC cells growth

**Fig. 4:**
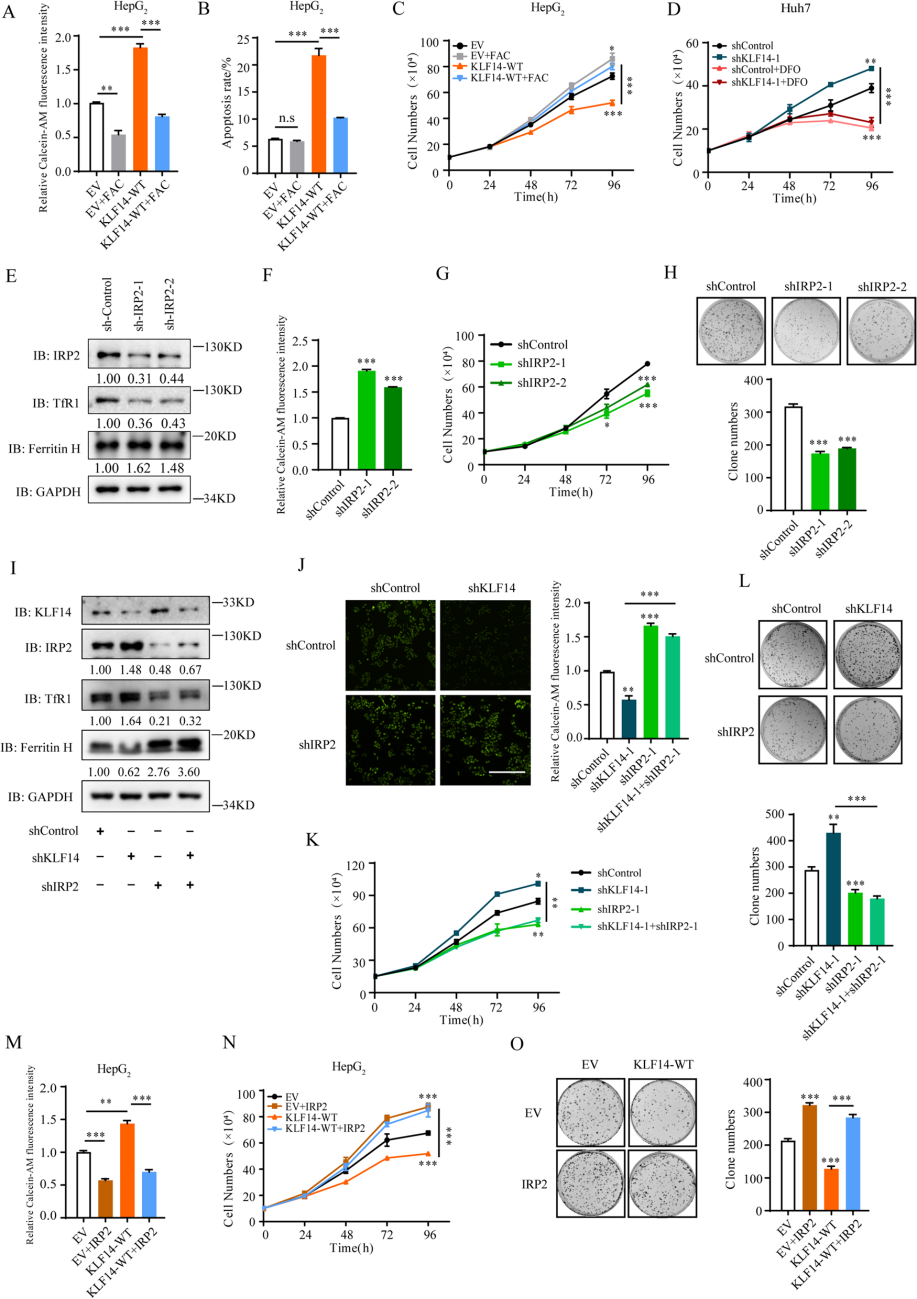
KLF14-mediated inhibition of HCC cells growth is dependent on iron and IRP2. **A** Relative cellular LIP content of HepG_2_ cells with KLF14 overexpressed were measured in standard media or media supplemented with 100 μM ferric ammonium citrate (FAC). **B** Cell apoptosis caused by KLF14 overexpression were reduced with FAC treatment. **C** Cell growth curve of HepG_2_ cells with KLF14 overexpressed in standard media or media supplemented 100 μM FAC for 4 days. **D** Cell growth curve of HepG_2_ cells with KLF14 silenced in standard media or media supplemented 100 μM DFO for 4 days. **E** Protein levels of IRP2, TfR1 and FH in IRP2 silenced cells were examined by western blotting. **F** Relative cellular LIP content of IRP2 silenced cells. **G** Cell growth curve of IRP2 silenced HepG_2_ cells. **H** Colony formation ability of HepG_2_ cells with IRP2 silenced. **I** Protein levels of IRP2, TfR1 and FH in KLF14 and/or IRP2 silenced cells were examined by western blotting. **J** The relative cellular LIP contents in KLF14 and/or IRP2 silenced cells were measured at 24 h (*left* panel) and 36 h (*right* panel). **K** Cell growth curve of KLF14 and/or IRP2 silenced HepG_2_ cells. **L** Colony formation ability of HepG_2_ cells with KLF14 and/or IRP2 silenced. **M** Relative cellular LIP content of KLF14 and/or IRP2 overexpressed cells. **N** Cell growth curve of KLF14 and/or IRP2 overexpressed HepG_2_ cells. **O** Colony formation ability was restored when IRP2 was overexpressed in KLF14 overexpressed cells. All results are presented as means ± SD from three independent experiments. Two-tailed unpaired Student’s T-tests were performed. n.s, not significant, *P < 0.05, **P < 0.01 and ***P < 0.001

Given that KLF14 induces cellular iron depletion and suppresses cell growth, we determined whether the modulation of cellular iron metabolism by IRP2 is important for the cell growth suppression in KLF14 overexpressed liver cancer cells. Cells were cultured in media with ferric ammonium citrate (FAC) to increase the intracellular iron content or Deferoxamine mesylate (DFO, an iron chelator) to induce cellular iron deficiency respectively. We found that FAC: (a) compensated the suppression of KLF14 on cellular LIP concentration (Fig. 4A); (b) rescued KLF14 induced apoptosis (Fig. 4B and Supplementary Fig. 3A); (c) caused cells to grow faster apparently compared with untreated cells; and (d) reversed full-length KLF14-induced cell growth suppression (Fig. 4C and Supplementary Fig. 3B). DFO dramatically inhibited liver cancer cells growth, providing the evidence of inducing cellular iron depletion is a promising method for HCC therapy [38, 39]. Meanwhile, DFO eliminated the growth-promoting effect of KLF14 knockdown cells (Fig. 4D and Supplementary Fig. 3C). Together, these results indicated that the amount of cellular LIP affects KLF14-medicated cell survival.

Next, we determined whether IRP2 is the functional target for KLF14-mediated cellular iron homeostasis and cells proliferation. We generated stable cell lines with IRP2 knockdown, and found that the expression of TFR1 was significantly reduced and the expression of FH was elevated (Fig. 4E). Subsequently, cellular LIP was significantly decreased (Fig. 4F). Moreover, cells grew more slowly, and caused significantly inhibition of clonogenic survival (Fig. 4G, H). These results indicated that inhibition of IRP2 induced cellular iron depletion and cell growth suppression. To further confirm the regulation of IRP2 by KLF14 is critical for KLF14-mediated cell growth, we inhibited the expression of IRP2 in KLF14-knockdown cells and overexpressed IRP2 in KLF14 overexpressed cells. Silence of IRP2 eliminated the effects of KLF14 knockdown in cellular iron concentration and cell proliferation (Fig. 4I-L). Furthermore, enhanced IRP2 expression restored the cellular LIP amount and cell growth in KLF14 overexpressed liver cancer cells (Fig. 4M-O and Supplementary Fig. 3D). Thus, the data supported the idea that IRP2 is the functional target of KLF14 in iron-related cell growth.

**Fig. 5:**
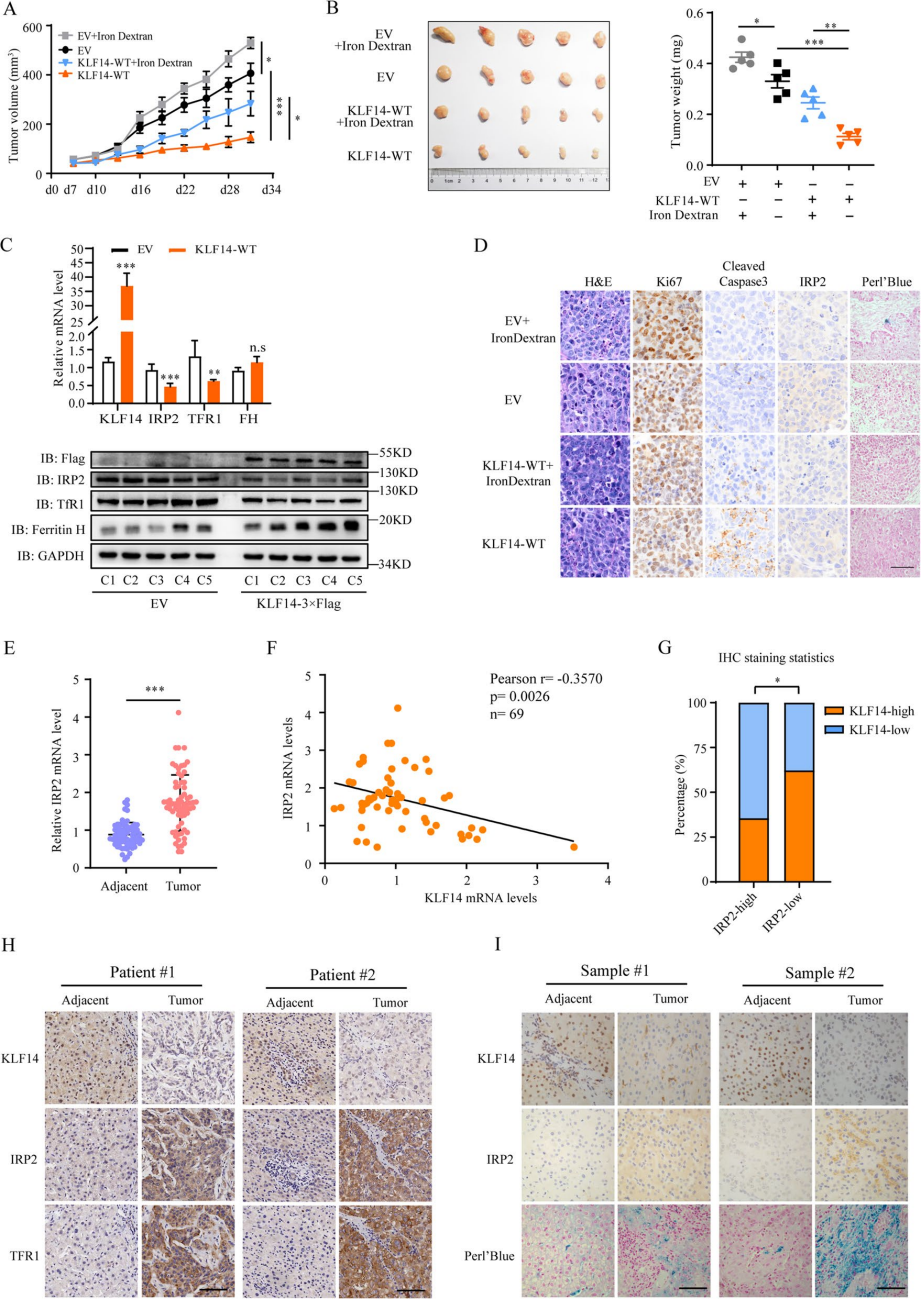
IRP2 is the target gene of KLF14 to regulate cellular iron content. **A** HepG_2_ cells with steady expression of KLF14-WT-3 × Flag or vector (control) were grafted into nude mice subcutaneously. The effects of KLF14 overexpression and/or iron supplementation on tumor growth in vivo was then assessed. *n* = 6. **B** Tumors were isolated and weight was measured. *n* = 6. **C** Relative mRNA and protein levels of KLF14, IRP2, TfR1, FH and GAPDH (as control) were examined by qRT-PCR and western blotting respectively. *n* = 6. **D** Immunohistochemistry staining for H&E, Perl’s Blue (iron), Ki67, cleaved caspase3 and IRP2 in tumor tissues isolated from mice. Scale bar, 50 μm. **E** Relative mRNA level of IRP2 in 69 paired tumor tissues and adjacent tissues of HCC. **F** The correlation analysis of KLF14 and IRP2 expression was conducted based on the mRNA levels of tumor tissues in HCC (*n* = 69). **G** Statistics of IHC staining displayed the percentages of HCC specimens with higher or lower KLF14 expression and corresponding IRP2 levels. **H** Representative images of KLF14 staining and corresponding IRP2/TfR1 staining were shown (scale bar, 100 μm). **I** Immunohistochemistry staining of KLF14, IRP2 and Perl’s Blue (iron) in HCC tumor and adjacent tissues (*n* = 4) were exhibited (scale bar, 50 μm). Two-tailed unpaired Student’s T-tests were performed. n.s, not significant, *P < 0.05, **P < 0.01 and ***P < 0.001

Given the function of KLF14 as observed in vitro, we then established a mouse xenograft model to investigate its effects on HCC in vivo. KLF14-WT-3 × Flag overexpressed HepG_2_ cells and control cells were subcutaneously injected into nude mice (Supplementary Fig. 3E). Intraperitoneal injection of iron dextran (250 μg per gram of body weight) and 0.9% NaCl were followed respectively when the tumor volume had reached approximately 100 mm^3^ [40]. Iron Dextra induced tumors growth and KLF14 suppressed the growth of tumors markedly. Notably, after the injection of iron dextran, the KLF14-WT-3 × Flag overexpressed tumors showed an obviously increase in growth, which partially rescued the suppression in vivo (Fig. 5A, B). Consistent with the data in vitro, the expression of IRP2 was downregulated in KLF14-overexpressed tumors, which resulted in reduced expression of TfR1 and upregulated protein level of FH (Fig. 5C). Perl’s Blue and Ki67 staining results showed that overexpression of KLF14 sharply reduced the cellular iron content and proliferation. Cleaved caspase 3 staining showed increased apoptosis in KLF14 overexpressed tumors. All of phenotypes were largely attenuated with injection of Iron Dextran (Fig. 5D and Supplementary Fig. 3F). Taken together, these data indicated that KLF14 regulates HCC growth by modulating iron homeostasis through the repression of IRP2 in vivo.

To determine clinical correlation of KLF14-IRP2 axis, we tested the expression of IRP2 in HCC patients. The results showed a higher expression of IRP2 in tumor tissues, which was correlated with worse outcomes in HCC (Fig. 5E and Supplementary Fig. 4A-C). A notable negative correlation between the mRNA levels of KLF14 and IRP2 was observed in tumor tissues (Fig. 5F). Co-staining of KLF14 and IRP2 in tissue microarray of HCC patients demonstrated that about 64.44% samples with lower KLF14 expression exhibited stronger IRP2 staining, while approximately 62.22% specimens with higher KLF14 expression showed weaker IRP2 staining (Fig. 5G, H). Obviously, the results illustrated that the expression of KLF14 and IRP2 are negatively correlated in HCC. Furthermore, TfR1 as a target of IRP2 was significantly upregulated in HCC and its high expression was related to poor prognosis (Supplementary Fig. 4D-F). The co-staining of KLF14, IRP2 and TfR1 in tissues microarray illustrated that the expression of TfR1 were positively correlated with IRP2 expression in HCC while negatively correlated with KLF14 expression (Supplementary Fig. 4G, H). Besides, Immunohistochemical images of the adjacent normal tissues and tumor tissues in HCC patients exhibited that the expression of KLF14 is lower in HCC tumor tissues together with higher IRP2 expression and iron concentration (Fig. 5I and Supplementary Fig. 4I). As KLF14 knockdown mediates an increase of iron concentration through elevating the expression of IRP2, we suggested KLF14 reduction contributes to IRP2 elevation and excessive iron in HCC tumor tissues. Together, these data strongly suggest that KLF14 regulates the expression of IRP2, which mediates subsequent cellular iron metabolism and HCC cells growth.

### KLF14 interacts with SIRT1 to silence IRP2

**Fig. 6:**
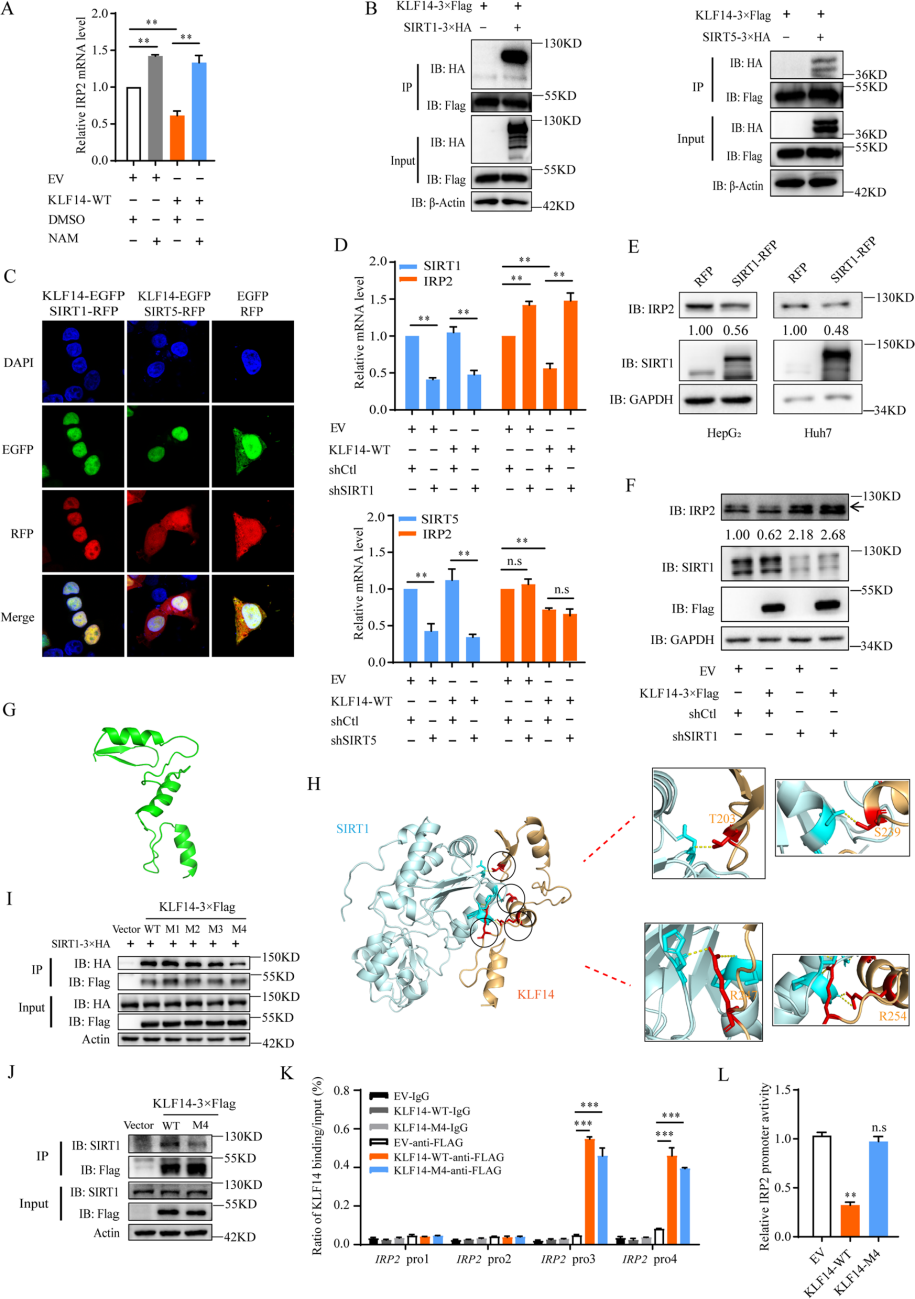
KLF14 suppresses the expression of IRP2 by recruiting SIRT1. **A** Relative mRNA level of IRP2 in KLF14 overexpressed cells and/or NAM treatment. **B** SIRT1–3 × HA or SIRT5–3 × HA was transfected into 293 T cells with KLF14-WT-3 × Flag for 48 h. Whole-cell lysates were immunoprecipitated with Flag beads and the co-precipitated HA was then detected. **C** Spatial analysis of KLF14 and SIRT1 or SIRT5 colocalization. SIRT1-RFP or SIRT5-RFP was transfected into 293 T cells with KLF14-EGFP for 48 h. Representative confocal images of KLF14 (green) and SIRT1/SIRT5 (red) in 293 T cells are shown. Scale bar, 10 μm. **D** Silencing of SIRT1 but not SIRT5 reduced the suppression of KLF14 on IRP2 mRNA level**. E** The protein level of IRP2 in SIRT1 overexpressed HepG_2_ and Huh7 cells. **F** The protein level of IRP2 in KLF14–3 × Flag overexpressed HepG_2_ cells with SIRT1 silenced. **G** Stimulated model of three zinc finger domain of IRP2 (green). **H** Stimulated model of the interaction between the zinc finger domain of IRP2 (yellow) and SIRT1 (blue), and the predicted binding sites of KLF14 (red). **I** KLF14–3 × Flag (WT), KL14-M1–3 × Flag (mut1: T203A), KL14-M2–3 × Flag (mut2: T203A + S239A), KL14-M3–3 × Flag (mut3: T203A + S239A + R247A) and KL14-M4–3 × Flag (mut4: T203A + S239A + R247A + R254A) transfected with SIRT1–3 × HA into 293 T cells for 48 h, whole-cell lysates were immunoprecipitated with Flag beads and the co-precipitated HA was detected. **J** KL14-M4–3 × Flag (mut4: T203A + S239A + R247A + R254A) was transfected into HepG_2_ cells, whole-cell lysates were immunoprecipitated with Flag beads and the co-precipitated SIRT1 was detected. **K** ChIP assay revealed significant enrichment of KLF14-M4 protein on the IRP2 promoter in HepG_2_ cells. **L** Mutation of KLF14 reversed the inhibition effect of KLF14-WT on IRP2 promoter activity. All the results are presented as means ± SD from three independent experiments. Two-tailed unpaired Student’s T-tests were performed. n.s, not significant, *P < 0.05, **P < 0.01 and ***P < 0.001

Recently, histone acetylation by acetylase has attracts much attention owing to its roles in gene transcription regulation, and deacetylase impair the promoter activity of target genes. The ability of deacetylase impairs the promoter activity of target genes through interacting with transcription factors has led to much interest [41, 42]. KLF14 has been demonstrated to represses the expression of TGFβRII via a co-repressor complex containing HDAC2 and mSin3A [43], indicating the possibility that KLF14 affects the histone acetylation of IRP2 through forming a complex with deacetylase. To further determine the mechanism of KLF14-mediated IRP2 inhibition, we treated KLF14 overexpressed cells with different deacetylases inhibitors, including type 1 and 2 HDAC family inhibitor trichostatin A (TSA) and type 3 HDAC family (Sirtuins) inhibitor Nicotinamide (NAM) [44, 45]. We found that NAM-treatment rescued the inhibition effects of KLF14 on IRP2, while rescue of KLF14 mediated IRP2 suppression showed minor changes in TSA-treatment group (Fig. 6A and Supplementary Fig. 5A). Previous studies have identified that transcription factors recruit SIRT1 to the promoter template with the deacetylation of template-associated histones H3/H4, in a manner that is unaffected by TSA but reversed by NAM [46]. We then hypothesized that KLF14 suppressed the expression of IRP2 through recruiting Sirtuins and thus reduced the histone acetylation of the IRP2 promoter. To determine which Sirtuin interacts with KLF14, we ectopically expressed SIRT1-SIRT7 and KLF14 plasmids. Then, we observed that exogenously expressed SIRT1 and SIRT5 interacted with KLF14 (Fig. 6B and Supplementary Fig. 5B-F). Moreover, immunofluorescence results showed that exogenously SIRT1 and SIRT5 co-localized with KLF14 (Fig. 6C). However, in KLF14 overexpressed cells, we found that knockdown of SIRT1, but not SIRT5, reversed the suppression effects of KLF14 upon IRP2 transcription (Fig. 6D and Supplementary Fig. 5G). Importantly, SIRT1 suppressed the expression of IRP2 in liver cancer cells at mRNA and protein levels (Fig. 6E and Supplementary Fig. 5H). And with SIRT1 silenced, the expression of IRP2 were significantly elevated (Supplementary Fig. 5I, J). Furthermore, silence of SIRT1 abolished the suppression effects of KLF14 on IRP2 expression, suggesting that KLF14 inhibits the expression of IRP2 dependent on SIRT1 (Fig. 6F). These results indicated that KLF14 and SIRT1 co-regulate the transcription of IRP2.

**Fig. 7:**
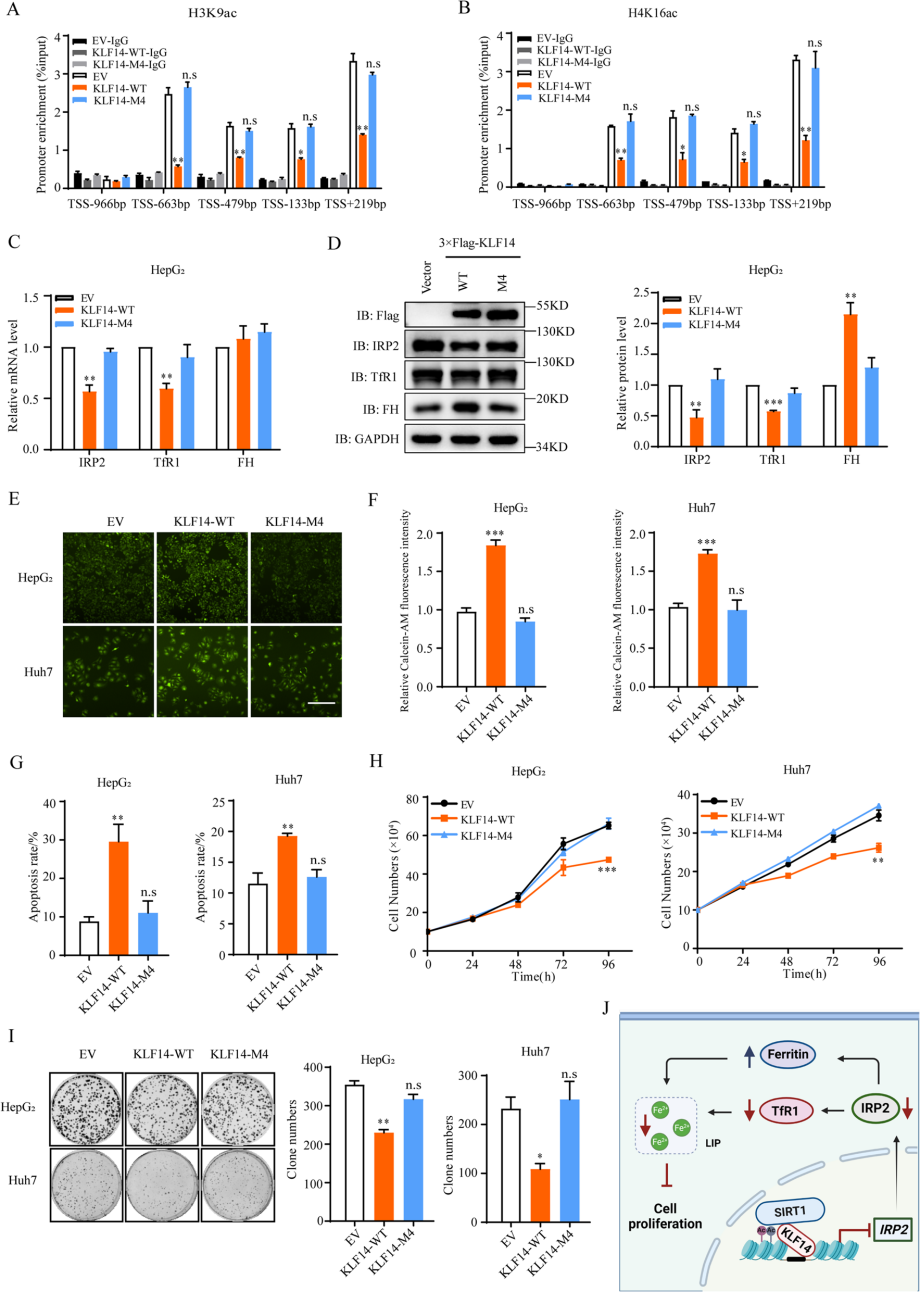
Mutation of KLF14 results in minimal change of HCC cells growth. **A, B** ChIP assay revealed downregulation effects of KLF14 on H3K9ac (**A**) and H4K16ac (**B**) of IRP2 promoter. KLF14-WT-3 × Flag and KLF14-M4–3 × Flag overexpressed HepG_2_ cells were harvested, the samples were immunoprecipitated with anti-H3K9ac and anti-H4K16ac antibody and the precipitated DNA fragments were subjected to qRT-PCR in the *IRP2* promoter regions. **C, D** Relative mRNA (**C**) and protein levels (**D**) of IRP2, TfR1, FH and GAPDH (control) in KLF14-M4–3 × Flag overexpressed HepG_2_ cells. **E, F** Relative cellular LIP content in KLF14-M4–3 × Flag overexpressed cell lines were measured at 24 h (**E**) and 36 h (**F**). **G** The cell apoptosis rate of KLF14-M4–3 × Flag overexpressed cells were investigated by flow cytometry. **H, I** Cell growth curve (**H**) and Colony formation ability (**I**) of KLF14-M4–3 × Flag overexpressed cells. **J** Schematic diagram depicted the working model of KLF14. It shows that KLF14 inhibits the transcription of IRP2 via recruiting SIRT1 which then causes TfR1 downregulation and ferritin upregulation on the basis of IRP-IREs system. This results in cellular iron deficiency and suppression of HCC cells growth. All the results are presented as means ± SD from three independent experiments. Two-tailed unpaired Student’s T-tests were performed. n.s, not significant, *P < 0.05, **P < 0.01 and ***P < 0.001

To further identify the relationships between KLF14, SIRT1 and IRP2, we simulated the structure of three zinc finger domains of KLF14 with a homologous template model of KLF4 (Fig. 6G). The molecular docking models of KLF14-SIRT1 and binding sites were predicated by using Z-DOCK 3.0.2 and GrammX prediction algorithms (Fig. 6H). Four KLF14 sites (Thr203, Ser239, Arg247 and Arg254) were predicted to be potential binding sites. With all four of these sites mutated to Alanine (KLF14-M4), the interaction between KLF14 and SIRT1 showed obvious reduction (Fig. 6I). In addition, the interaction between KLF14 and endogenous SIRT1 almost vanished in KLF14-M4 overexpressed HepG_2_ cells (Fig. 6J), which further demonstrated that KLF14’s ability to recruit SIRT1 is largely reduced with the binding sites mutated. KLF14-M4 showed an obvious binding on the promoter of IRP2 (Fig. 6K). However, KLF14-M4 mutant eliminated the inhibition effects of KLF14 on IRP2 promoter luciferase activity (Fig. 6L). ChIP assay was also conducted to detect the acetylation level of H3K9 and H4K16, which are the key de-acetylation sites affected by SIRT1 [47, 48], around the target region of IRP2 promoter. The acetylation around the target region in the IRP2 promoter were obviously downregulated, however, such changes did not occur in mutated KLF14 (KLF14-M4) overexpressed cells (Fig. 7A, B). Taken together, these results suggested that KLF14 recruits SIRT1 to reduce the histone acetylation and leading to suppression of IRP2 transcription.

To further confirm that KLF14 inhibits the growth of liver cancer cells through interacting with SIRT1, we performed mutated KLF14 (KLF14-M4) stably overexpressed HCC cells. There was no significant changes of the expression of IRP2 and other iron related proteins upon mutated KLF14 overexpression (Fig. 7C, D and Supplementary Fig. 6A-C). Mutated KLF14 also showed negligible effects on the intracellular LIP concentration and cell apoptosis (Fig. 7E-G and Supplementary Fig. 6D). Moreover, mutation of KLF14 eliminated the suppression effects of full-length KLF14 on the growth and clonogenic formation of liver cancer cells (Fig. 7H, I). These data suggested that KLF14 regulates the proliferation of HCC cells through suppressing IRP2 which is dependent on the recruitment of SIRT1. In conclusion, KLF14 inhibits the transcription of IRP2 via recruiting SIRT1, which then causes TfR1 downregulation and ferritin upregulation, resulting in cellular iron deficiency and suppression of HCC cells growth (Fig. 7J).

### Fluphenazine activates KLF14 and impairs the cellular iron homeostasis

Accumulated data has shown that drugs targeting the cellular iron metabolism exert anti-proliferative effects in cancer cells, such as iron chelators [49–51]. Our results suggested that KLF14 regulates the cellular iron homeostasis by repressing the expression of IRP2, supporting the idea that activation of endogenous *KLF14* could decrease intracellular iron concentration and suppress the proliferation of HCC. A previous study demonstrated fluphenazine as one of the compounds that activates *KLF14*-luc activity [52]. Although the liver cancer-combating properties of fluphenazine have been identified [53], its detailed molecular mechanism and whether it plays role in iron metabolism remain unclear.

**Fig. 8:**
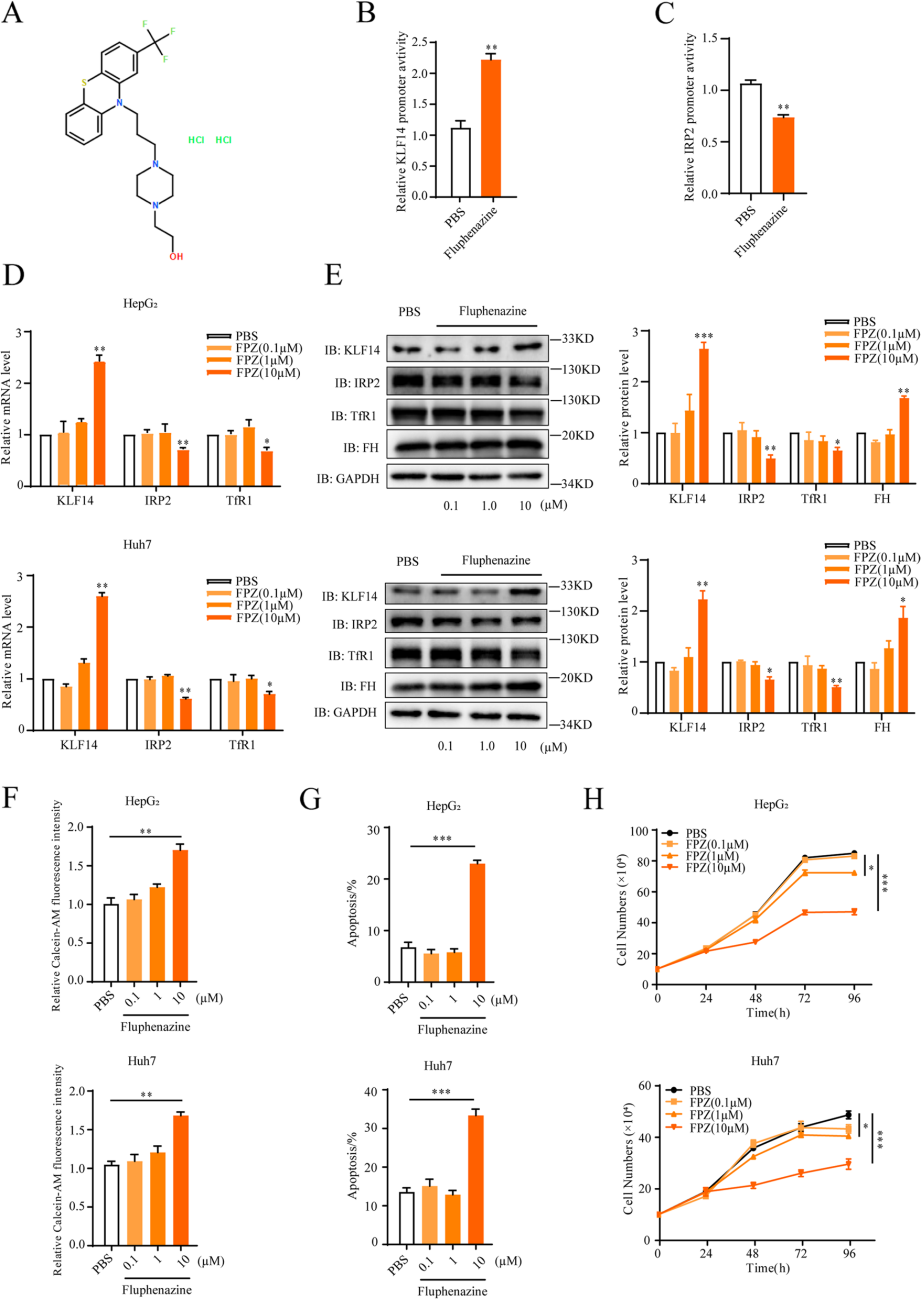
Fluphenazine is an activator of KLF14 and inhibits HCC cells growth. **A** The chemical structure of Fluphenazine Hydrochloride. **B** The luciferase activity of reporters was analyzed in HepG_2_ cells transfected with pGL4-*KLF14*-luc constructs after 48 hours treatment with 10 μM Fluphenazine Hydrochloride or PBS. **C** HepG_2_ cells were transfected with pGL4-*IRP2*-luc plasmids for 12 hours and incubated with 10 μM Fluphenazine Hydrochloride or PBS for 48 hours. *IRP2* promoter luciferase activity was then examined. **D, E** HepG_2_ and Huh7 cells were treated with PBS or Fluphenazine Hydrochloride at the indicated dosage for 48 h, mRNA (**D**) and protein levels (**E**) of KLF14 and IRP2 were examined by qRT-PCR and western blotting respectively. **F** Relative cellular LIP content of HepG_2_ and Huh7 cells were measured with the treatment of PBS or Fluphenazine Hydrochloride at the indicated dosage for 36 h. **G** The cell apoptosis rate of HepG_2_ and Huh7 cells with incubation of PBS or Fluphenazine Hydrochloride at the indicated dosage for 48 h were investigated by flow cytometry. **H** Cell growth curve of HepG_2_ and Huh7 cells were measured in media supplemented with PBS or Fluphenazine Hydrochloride at the indicated dosage for 4 days. All the results are presented as means ± SD from three independent experiments. Two-tailed unpaired Student’s T-tests were performed. n.s, not significant, *P < 0.05, **P < 0.01 and ***P < 0.001

In this study, we firstly confirmed that fluphenazine (10 μM) markedly elevated the promoter luciferase activity of KLF14 for more than 2-fold after incubation for 48 hours (Fig. 8A, B). At the same time, the promoter activity of IRP2 was inhibited by fluphenazine (Fig. 8C). Next, we demonstrated that fluphenazine induced the expression of KLF14 in HepG_2_ and Huh7 cells. Moreover, fluphenazine treatment caused suppression of IRP2 together with the reduction of TfR1/DMT1 and upregulation of ferritin (Fig. 8D, E and Supplementary Fig. 7A). As a result, the cellular iron concentration was decreased obviously with the treatment of fluphenazine (Fig. 8F). Furthermore, incubation of fluphenazine induced HCC cells apoptosis and HCC cells growth suppression (Fig. 8G, H and Supplementary Fig. 7B). These findings identified fluphenazine as an activator of KLF14, and it caused impairment to cellular iron metabolism, and suppressed cell growth of HCC cells.

### Fluphenazine suppresses the iron-related cell growth of HCC via activating KLF14

**Fig. 9:**
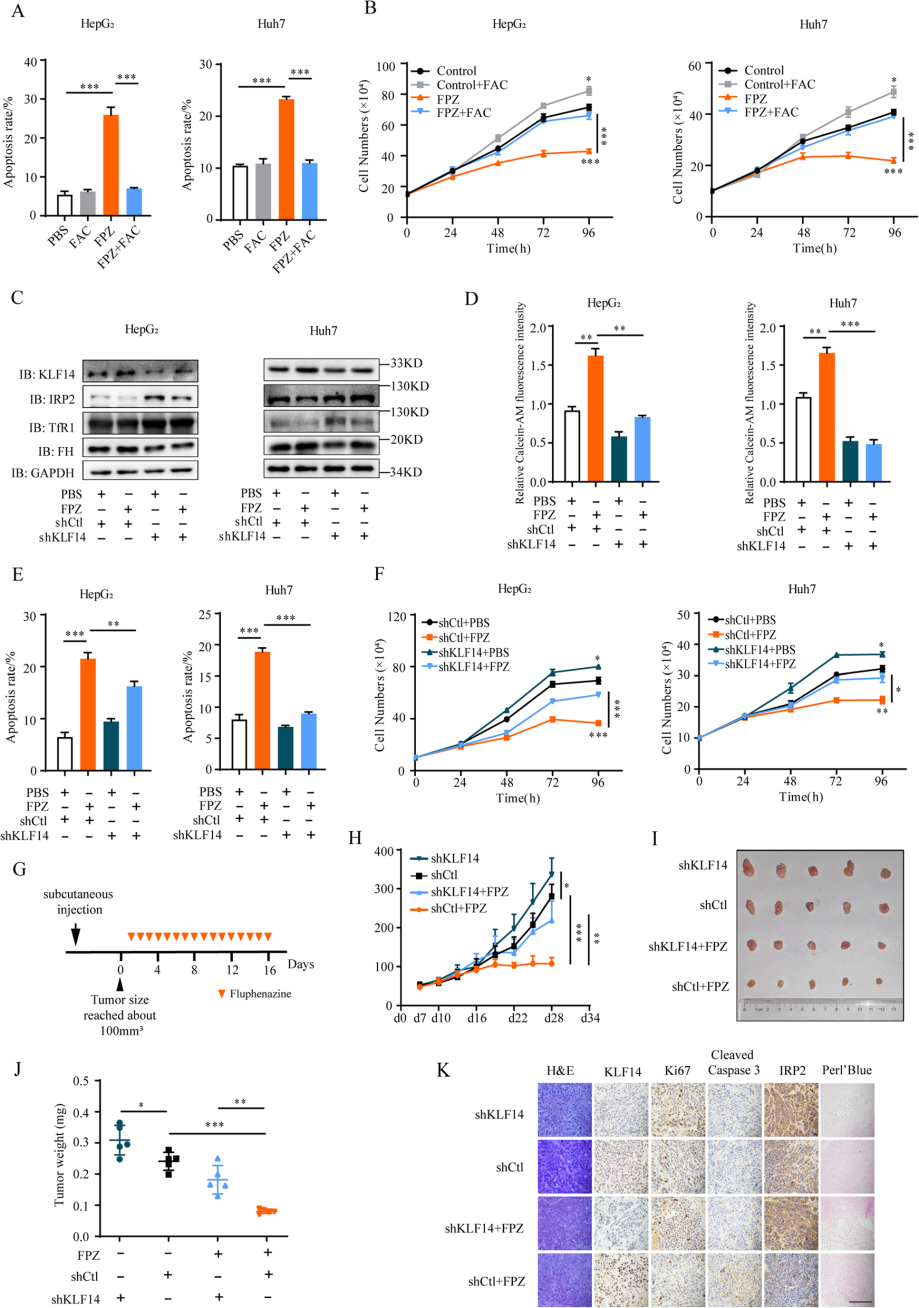
Fluphenazine-mediated inhibition of HCC growth is dependent on iron and KLF14. **A** Cell apoptosis induced by fluphenazine (10 μM) were decreased with FAC (100 μM) treatment. **B** Cell growth curve of HepG_2_ and Huh7 cells were measured in media supplemented with PBS, fluphenazine (10 μM), or their combination with FAC for 4 days. **C** Western blotting tested the expression of KLF14, IRP2, TfR1 and FH in KLF14-silenced cells with fluphenazine (10 μM) treatment. **D** KLF14-silenced cells were incubated with 10 μM fluphenazine for 36 h. The relative cellular LIP content were measured using calcein-AM assay. **E** Silencing KLF14 reduced the cell apoptosis induced by fluphenazine (10 μM). **F** Cell growth curve of KLF14-silenced HepG_2_ and Huh7 cells were measured in media supplemented with PBS or fluphenazine (10 μM) for 4 days. **G** HepG_2_ cells were grafted into nude mice subcutaneously. When the tumor volume reached approximately 100 mm^3^, intraperitoneal injection of fluphenazine (8 mg/kg) was followed before harvesting solid tumors. Xenograft tumors were analyzed at indicated time points. *n* = 6. **H** Tumor volume was measured starting 7 days after inoculation. **I, J** Tumors were isolated and weight was measured. *n* = 6. **K** Immunohistochemistry staining for H&E, Perl’s Blue (iron), Ki67, cleaved caspase3, KLF14 and IRP2 in tumor tissues isolated from mice (scale bar 50 μm). All the results are presented as means ± SD from three independent experiments. Two-tailed unpaired Student’s T-tests were performed. n.s, not significant, *P < 0.05, **P < 0.01 and ***P < 0.001

Our data demonstrated that KLF14 mediated iron-related cell growth in HCC cells. As fluphenazine activated the expression of KLF14, and suppressed the growth of HCC cells, we considered whether fluphenazine-mediated phenotypes rely on iron. To test this, cells were grown in media containing fluphenazine and FAC. Interestingly, the cell apoptosis induced by fluphenazine alone were significantly reversed (Fig. 9A and Supplementary Fig. 8A). Besides, FAC was clearly associated with an apparent increase in cellular proliferation and largely attenuated the suppression of fluphenazine on cell growth (Fig. 9B). These results suggested that the suppression of fluphenazine on the proliferation of HCC cells was largely dependent on iron.

To further examine if KLF14 deficiency block fluphenazine-induced suppression of IPR2 expression and cellular LIP concentration, KLF14-silenced liver cancer cells and control cells were treated with fluphenazine for 48 h. We found a significant decrease in IRP2 expression in fluphenazine-treated control cells, though the negative effect was lessened in fluphenazine-treated KLF14-silenced cells. Otherwise, a reduction of TfR1 expression and an increase of ferritin expression were measured in fluphenazine-treated control cells, but not in fluphenazine-treated KLF14-silenced cells (Fig. 9C). Subsequently, fluphenazine-treated control cells showed a significant reduction of cellular LIP content compared with fluphenazine-treated KLF14-silenced cells (Fig. 9D). Moreover, cell apoptosis and growth inhibition existed in fluphenazine-treated control cells, and all of these effects were largely alleviated in fluphenazine treated KLF14-silenced cells (Fig. 9E, F and Supplementary Fig. 8B). These data illuminated that the administration of fluphenazine impaired cellular iron metabolism and suppressed the cell growth is partly dependent on KLF14.

To determine whether the suppression effect of fluphenazine on the development of HCC depends on the activation of KLF14 in vivo, we performed a mouse xenograft model via the subcutaneous injection of control or KLF14-silenced HepG2 cells in nude mice (Fig. 9G). Upon tumor volume reaching around 100 mm^3^, daily intraperitoneal injection of fluphenazine (8 mg/kg) or 0.9% NaCl were followed until harvesting solid tumors [54]. Interestingly, we observed that knockdown of KLF14 promoted the development of HCC in vivo. And injection of fluphenazine significantly inhibited the growth of tumor while silencing of KLF14 attenuated the suppression effect (Fig. 9H, I, J), which were consistent with results the in vitro. IRP2 expression was increased in KLF14-silenced tumors and fluphenazine treated KLF14-silenced tumors, which indicated that knockdown of KLF14 alleviated the suppression effect of fluphenazine on IRP2 expression (Fig. 9K and Supplementary Fig. 8C). Also, fluphenazine-treated tumors showed enhanced iron deficiency, apoptosis and inhibition of proliferation compared with fluphenazine-treated KLF14-silenced tumors (Fig. 9K and Supplementary Fig. 8D-F). Collectively, our results provide a novel therapy for HCC by fluphenazine administration which activated KLF14 causing iron deficiency and then suppresses cell growth in vitro and in vivo.

## Discussion

Since the identification of KLF14 as a member of the SP/XKLF transcription factor family [55], its role in tumorigenesis has drawn increasing attention [56]. A previous study has reported that lncRNA DGCR5 could inhibit HCC via miR-346/KLF14 axis [57]. However, the role of KLF14 in HCC and the specific mechanism of action are still unclear. In the present study, we firstly demonstrated that KLF14 exerts its anti-HCC function by inhibiting IRP2 and impairing cellular iron homeostasis. KLF14 interacts with SIRT1 contributing to the reduction of IRP2 transcriptional activity, which suggests a transcriptional repressor role of KLF14 in HCC. Notedly, we identified a small molecular drug, fluphenazine, as an activator of KLF14. Fluphenazine administration impairs iron homeostasis and showed anti-HCC effect.

Cancer cells have higher metabolic iron demand to sustain their division, growth, and survival [58]. IRP2 plays a key role in regulating cellular iron homeostasis. Both in vitro and in vivo assays have confirmed that IRP2 drives liver cancer cell growth [59], and its protein abundance is associated with a poor clinical outcome in these HCC patients [60]. IRP2 could be specially recognized by E3 ubiquitin ligase FBXL5 and then be degraded through ubiquitination when the cellular iron level is high [61]. Hepatic FBXL5 deficiency induced IRP2 accumulation was able to induce iron overload and promote carcinogenesis, suggesting that targeting IRP2 may be effective to alleviate hepatocellular iron overload and IRP2 could be a potential candidate for HCC treatment [60]. In this study, we firstly illustrated that KLF14 suppresses the expression of IRP2 through inhibiting the transcription, which is different from FBXL5. Meanwhile, KLF14 overexpression led to cellular LIP concentration reduction and cell growth inhibition. These data were consistent with the results that IRP2 knockout mice displaying dysregulation of iron metabolism with iron deficiency [62]. The study further expands our understanding of the upstream transcription regulatory mechanism of IRP2 by KLF14 as well as the crucial role of IRP2 in the tumor cell iron metabolism and it would be helpful for the understanding of the progression of HCC.

KLF14 has a large CpG island that spans almost its entire open reading frame, which is different from other KLFs [63]. The regions enriched with GC regulate the transcription of downstream genes via interacting with co-activators and suppressors [13]. KLF14 has been demonstrated to represses the expression of TGFβRII via a co-repressor complex containing HDAC2 and mSin3A [43]. Our findings demonstrated KLF14 as a repressor of IRP2 in HCC cells. KLF14 decreased histone acetylation through recruiting SIRT1 to the IRP2 promoter. KLF14-mediated the suppression of IRP2 was dependent on SIRT1, which suggests a KLF14-related epigenetic mechanism in the regulation of iron homeostasis and tumor growth in HCC.

Iron deprivation has been identified to suppress HCC growth. For example, the iron chelator TSC24 has been found to reduce liver cancer cell iron content and suppress its proliferation [23]. Similar results have also been obtained by targeting IRP2 [60]. As reported, cisplatin binds to IRP2 and mediates inactivation of IRP2, which causes the downregulation of TfR1 and the upregulation of ferritin, leading to sustained intracellular iron deficiency and suppression of cancer cells growth [64]. In this regard, we propose the KLF14–IRP2 axis as a potentially valuable therapeutic target for human HCC. Given that complete loss of IRP2 gives rise to microcytic anemia or neurodegeneration in mice [65, 66], which indicates a suitable level of IRP2 downregulation would be benefit for clinical treatment. Fluphenazine, is usually typified as an antipsychotic drug [67, 68]. Over recent years, fluphenazine has been studied in many types of cancers and shown to exhibits anti-cancer properties [69–73]. Here, we first discovered fluphenazine, activates KLF14, inhibits the expression of IRP2, impairs cellular iron metabolism and leads to the suppression of the liver cancer cell growth in vitro and *vivo*. Our research in HCC cells further suggests its high potential as an anti-tumor agent as an activator of KLF14. Fluphenazine administration reduced IRP2 expression and cellular LIP contents. Although we cannot exclude the possibility that the unknown target genes of fluphenazine contribute to the suppression effect of HCC, our combined biochemical, cellular, molecular analyses on HCC cells in vitro and mouse models in vivo strongly suggest KLF14 as a major downstream target to mediate the function of fluphenazine on HCC. It is still unclear how fluphenazine activates KLF14 and this will provide an interesting focus for future studies.

## Conclusions

In summary, our study firstly demonstrated that KLF14 acts as a tumor suppressor in HCC and inhibits the HCC cells growth through reducing cellular LIP contents by suppressing IRP2. The KLF14-IRP2 axis is clinically and functionally associated with HCC. Importantly, we identified fluphenazine as an anti-HCC compound that suppressed the growth of HCC cells through activating KLF14 and downregulating the cellular LIP concentration. These findings provide a critical role of KLF14 in HCC development and convincing evidence to support KLF14 as a novel therapeutic target for HCC.

## Supplementary Information

**Additional file 1: Supplementary Fig. 1. (A, B)** qRT-PCR was used to test the mRNA level of KLF14 in KLF14 overexpressed or silenced HepG_2_ and Huh7 cells. Data represent means ± SD, ***P < 0.001.

**Additional file 2: Supplementary Fig. 2. (A)** HepG_2_ cells were transfected with KLF14-WT-3 × Flag, KLF14-ΔZF1–3 × Flag, KLF14-ΔZF2–3 × Flag, KLF14-ΔZF3–3 × Flag, KLF14-ΔZF123–3 × Flag or vector for 48 h, and the mRNA level of IRP2 was measured by qRT-PCR. **(B)** The protein expression of DMT1 in KLF14-WT-3 × Flag or KLF14-ΔZF3–3 × Flag overexpressed HepG_2_ and Huh7 cells. **(C)** The protein expression of DMT1 in KLF14 silenced HepG_2_ and Huh7 cells. **(D)** Cell apoptosis of KLF14-WT-3 × Flag and KLF14-ΔZF3–3 × Flag overexpressed cells were investigated by flow cytometry. Data represent means ± SD, n.s, not significant, **P < 0.01.

**Additional file 3: Supplementary Fig. 3. (A)** KLF14 overexpressed HepG_2_ cells were treated with FAC (100 μM) for 48 h, then cell apoptosis was investigated by flow cytometry. **(B)** Cell growth curve of Huh7 cells with KLF14 overexpressed in standard media or media supplemented 100 μM FAC for 4 days. **(C)** Cell growth curve of HepG_2_ cells with KLF14 silenced in standard media or media supplemented 100 μM DFO for 4 days. **(D)** Cell growth curve of Huh7 cells with KLF14 and/or IRP2 overexpressed. **(E)** Schematic diagram of the treatment regimen applied to mice subcutaneously implanted with KLF14-WT-3 × Flag overexpressed HepG_2_ cells and control cells. Mice were administered 0.9% NaCl or Iron Dextran daily via intraperitoneal injection (*n* = 6). **(F)** The relative intensities of IHC staining or percentage of positive cells in tumors isolated from KLF14-overexpressed or/and iron supplementation group were quantified by Image J (version w.8.0). Data represent means ± SD, n.s, not significant, **p < 0.01, ***p < 0.001.

**Additional file 4: Supplementary Fig. 4. (A)** Relative mRNA level of IRP2 in 69 paired tumor tissues and adjacent tissues of HCC. **(B)** Representative IHC staining of IRP2 in HCC tumor and adjacent tissues (*n* = 90) were exhibited (scale bar, 100 μm). **(C)** Kaplan-Meier analyses were conducted to evaluate the overall survival according to the expression of IRP2 in HCC tissue chip. **(D)** Relative mRNA level of TfR1 in 69 paired tumor tissues and adjacent tissues of HCC. **(E)** Representative IHC staining of TfR1 in HCC tumor and adjacent tissues were exhibited (scale bar, 100 μm). **(F)** Kaplan-Meier analyses were conducted to evaluate the overall survival according to the expression of TfR1 in HCC tissue chip. **(G)(H)** Statistics of IHC staining displayed the percentages of HCC specimens with higher or lower IRP2/KLF14 expression and corresponding TfR1 levels. **(I)** Immunohistochemistry staining of KLF14, IRP2 and Perl’s Blue (iron) in HCC tumor and adjacent tissues (*n* = 4) were exhibited (scale bar, 50 μm). Two-tailed unpaired Student’s T-tests were performed. *P < 0.05, and ***P < 0.001.

**Additional file 5: Supplementary Fig. 5. (A)** Relative mRNA level of IRP2 in KLF14 overexpressed cells and/or TSA treatment. (**B)** SIRT2, 3, 4, 6, 7–3 × HA and KLF14-WT-3 × Flag were transfected into 293 T cells for 48 h, whole-cell lysates were immunoprecipitated with Flag beads and the co-precipitated HA was detected. **(C)** Silencing of SIRT1 but not SIRT5 reduced the suppression effects of KLF14 on IRP2 promoter activity. **(E)** Relative mRNA level of IRP2 in SIRT1 overexpressed cells. **(F)** Relative mRNA level of IRP2 in cells with SIRT1 silenced. (G) The protein level of IRP2 in cells with SIRT1 silenced. Data represent means ± SD, n.s, not significant, *P < 0.05, **p < 0.01, ***p < 0.001.

**Additional file 6: Supplementary Fig. 6. (A)** Relative mRNA levels of IRP2, TfR1, FH and GAPDH (control) in KLF14-M4–3 × Flag overexpressed Huh7 cells. **(B**-**C)** The protein levels of IRP2, TfR1, FH and GAPDH (control) in KLF14-M4–3 × Flag overexpressed Huh7 cells. **(D)** Cell apoptosis of KLF14-M4–3 × Flag overexpressed cells were investigated by flow cytometry. Data represent means ± SD, n.s, not significant, *P < 0.05, **p < 0.01, ***p < 0.001.

**Additional file 7: Supplementary Fig. 7. (A)** The protein levels of DMT1 in HepG_2_ and Huh7 cells with fluphenazine treatment. **(B)** Cell apoptosis of HepG_2_ and Huh7 cells with incubation of PBS or fluphenazine at the indicated dosage for 48 h were investigated by flow cytometry.

**Additional file 8: Supplementary Fig. 8. (A)** Cell apoptosis of HepG_2_ and Huh7 cells with incubation of PBS, 10 μM Fluphenazine or the combination with 100 μM FAC for 48 h, were investigated by flow cytometry. **(B)** KLF14-silenced HepG_2_ and Huh7 cells were treated with PBS or 10 μM Fluphenazine for 48 h, then cell apoptosis was investigated by flow cytometry. **(C**-**F)** The relative intensities of IHC staining or percentage of positive cells in tumor tissues isolated from mice were quantified by Image J (version w.8.0). Data represent means ± SD, n.s, not significant, **p < 0.01, ***p < 0.001.

**Additional file 9: Table S1.** Primers for qRT-PCR. **Table S2.** Primers for ChIP-qPCR. **Table S3.** Correlation between KLF14 expression and clinicopathologic features in patients with HCC.

## Data Availability

All data associated with this study are present in the paper or the Supplementary Materials.

## Abbreviations

KLF14: Krüpple-like factor 14
HCC: Hepatocellular carcinoma
IRP2: Iron-responsive element-binding protein 2
TfR1: Transferrin receptor protein 1
FH: Ferritin heavy chain
DMT1: Divalent metal transporter 1
FPN: Ferroportin
LIP: Liable iron pool
SIRT1: Sirtuin 1
FAC: Ferric ammonium citrate
DFO: Desferoxamine
FPZ: Fluphenazine
