# Supplementary figures and images for: KLF14 regulates the growth of hepatocellular carcinoma cells via its modulation of iron homeostasis through the repression of iron-responsive element-binding protein 2
Source: J Exp Clin Cancer Res. 2023 Jan 5;42:5. doi: 10.1186/s13046-022-02562-4 (PMC9814450; doi:10.1186/s13046-022-02562-4)

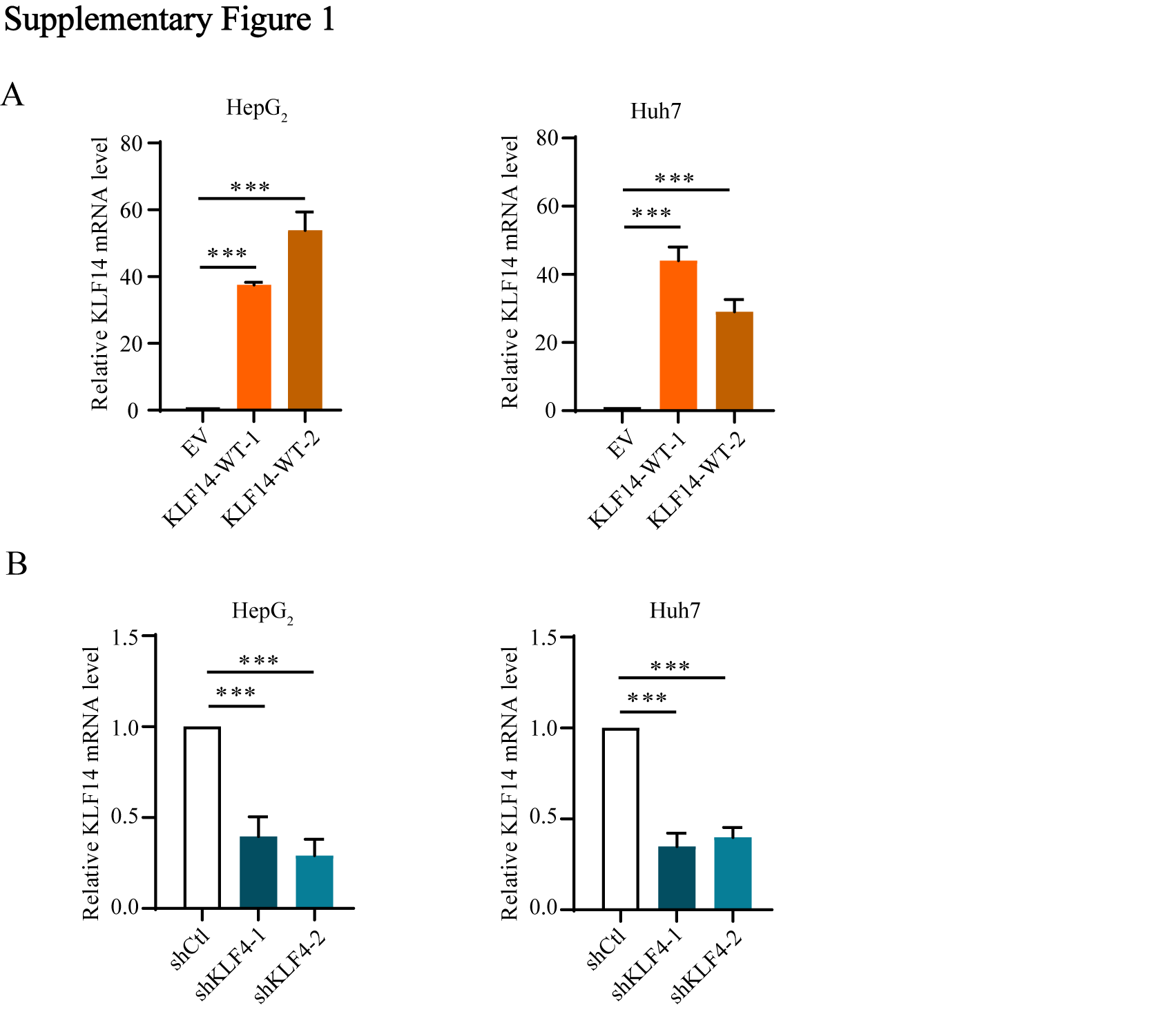

Supplement: Supplementary file 1 — Additional file 1: Supplementary Fig. 1. (A, B) qRT-PCR was used to test the mRNA level of KLF14 in KLF14 overexpressed or silenced HepG2 and Huh7 cells. Data represent means ± SD, ***P < 0.001. [file 13046_2022_2562_MOESM1_ESM.tif]

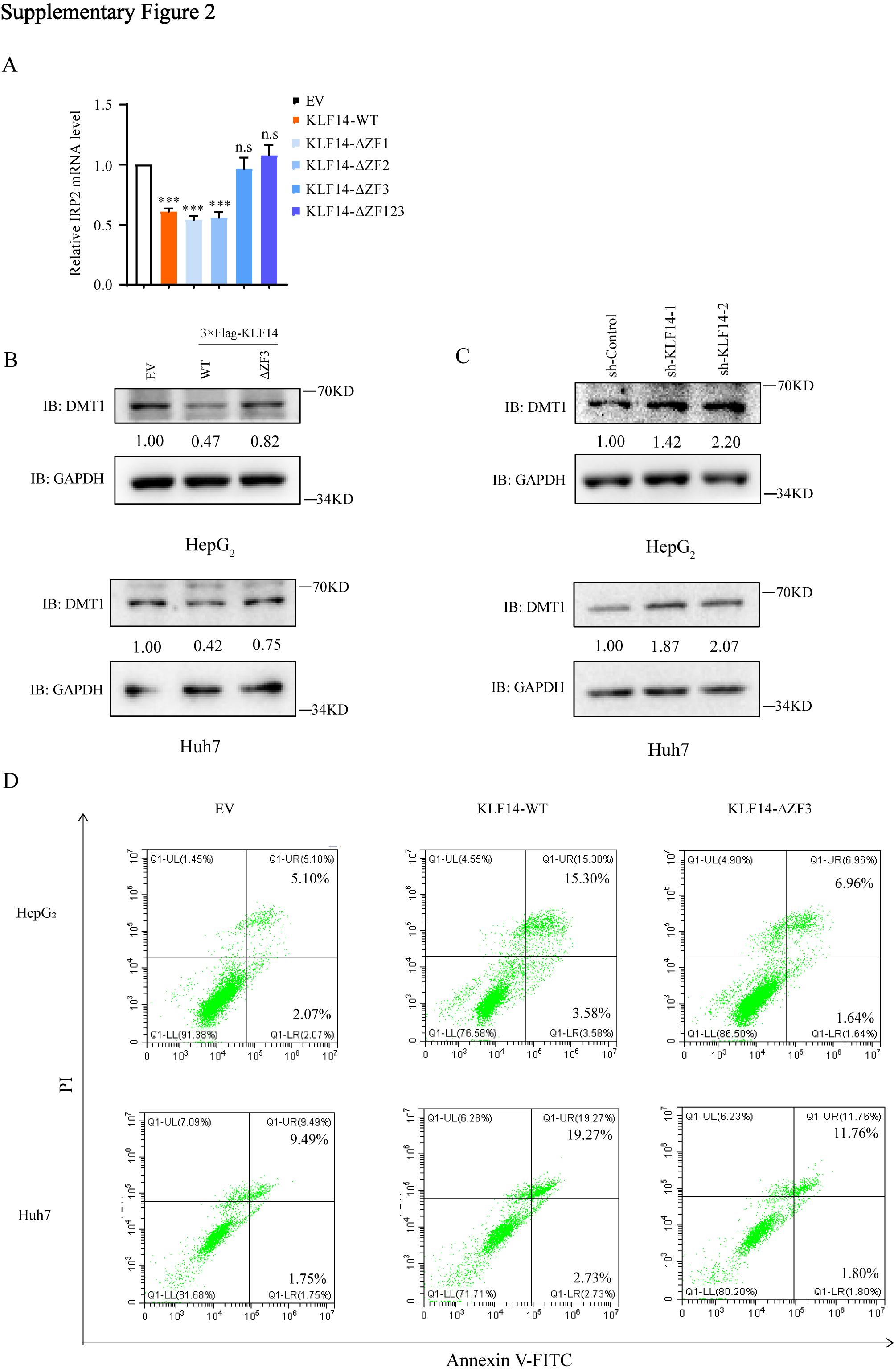

Supplement: Supplementary file 2 — Additional file 2: Supplementary Fig. 2. (A) HepG2 cells were transfected with KLF14-WT-3 × Flag, KLF14-ΔZF1–3 × Flag, KLF14-ΔZF2–3 × Flag, KLF14-ΔZF3–3 × Flag, KLF14-ΔZF123–3 × Flag or vector for 48 h, and the mRNA level of IRP2 was measured by qRT-PCR. (B) The protein expression of DMT1 in KLF14-WT-3 × Flag or KLF14-ΔZF3–3 × Flag overexpressed HepG2 and Huh7 cells. (C) The protein expression of DMT1 in KLF14 silenced HepG2 and Huh7 cells. (D) Cell apoptosis of KLF14-WT-3 × Flag and KLF14-ΔZF3–3 × Flag overexpressed cells were investigated by flow cytometry. Data represent means ± SD, n.s, not significant, **P < 0.01. [file 13046_2022_2562_MOESM2_ESM.tif]

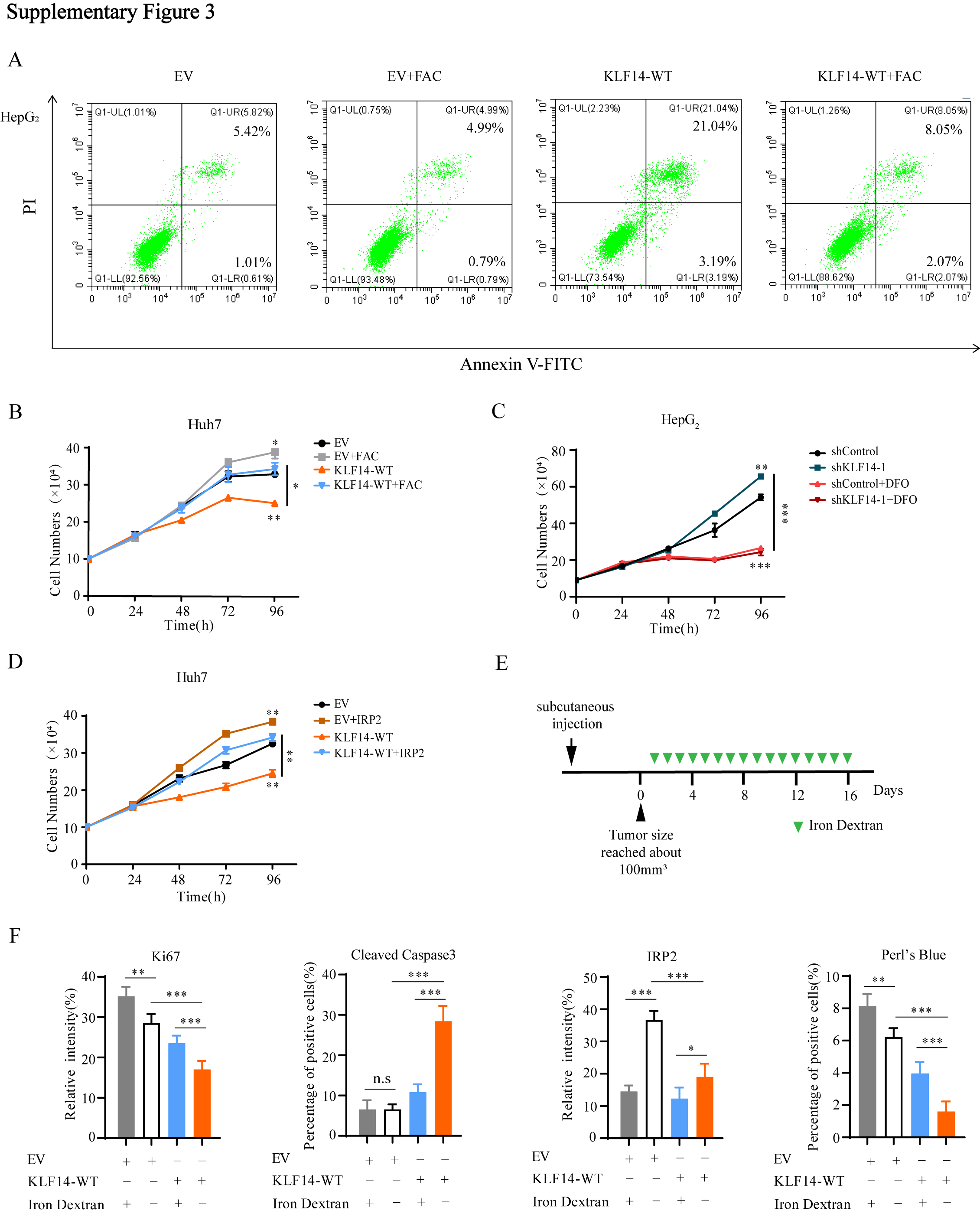

Supplement: Supplementary file 3 — Additional file 3: Supplementary Fig. 3. (A) KLF14 overexpressed HepG2 cells were treated with FAC (100 μM) for 48 h, then cell apoptosis was investigated by flow cytometry. (B) Cell growth curve of Huh7 cells with KLF14 overexpressed in standard media or media supplemented 100 μM FAC for 4 days. (C) Cell growth curve of HepG2 cells with KLF14 silenced in standard media or media supplemented 100 μM DFO for 4 days. (D) Cell growth curve of Huh7 cells with KLF14 and/or IRP2 overexpressed. (E) Schematic diagram of the treatment regimen applied to mice subcutaneously implanted with KLF14-WT-3 × Flag overexpressed HepG2 cells and control cells. Mice were administered 0.9% NaCl or Iron Dextran daily via intraperitoneal injection (n = 6). (F) The relative intensities of IHC staining or percentage of positive cells in tumors isolated from KLF14-overexpressed or/and iron supplementation group were quantified by Image J (version w.8.0). Data represent means ± SD, n.s, not significant, **p < 0.01, ***p < 0.001. [file 13046_2022_2562_MOESM3_ESM.tif]

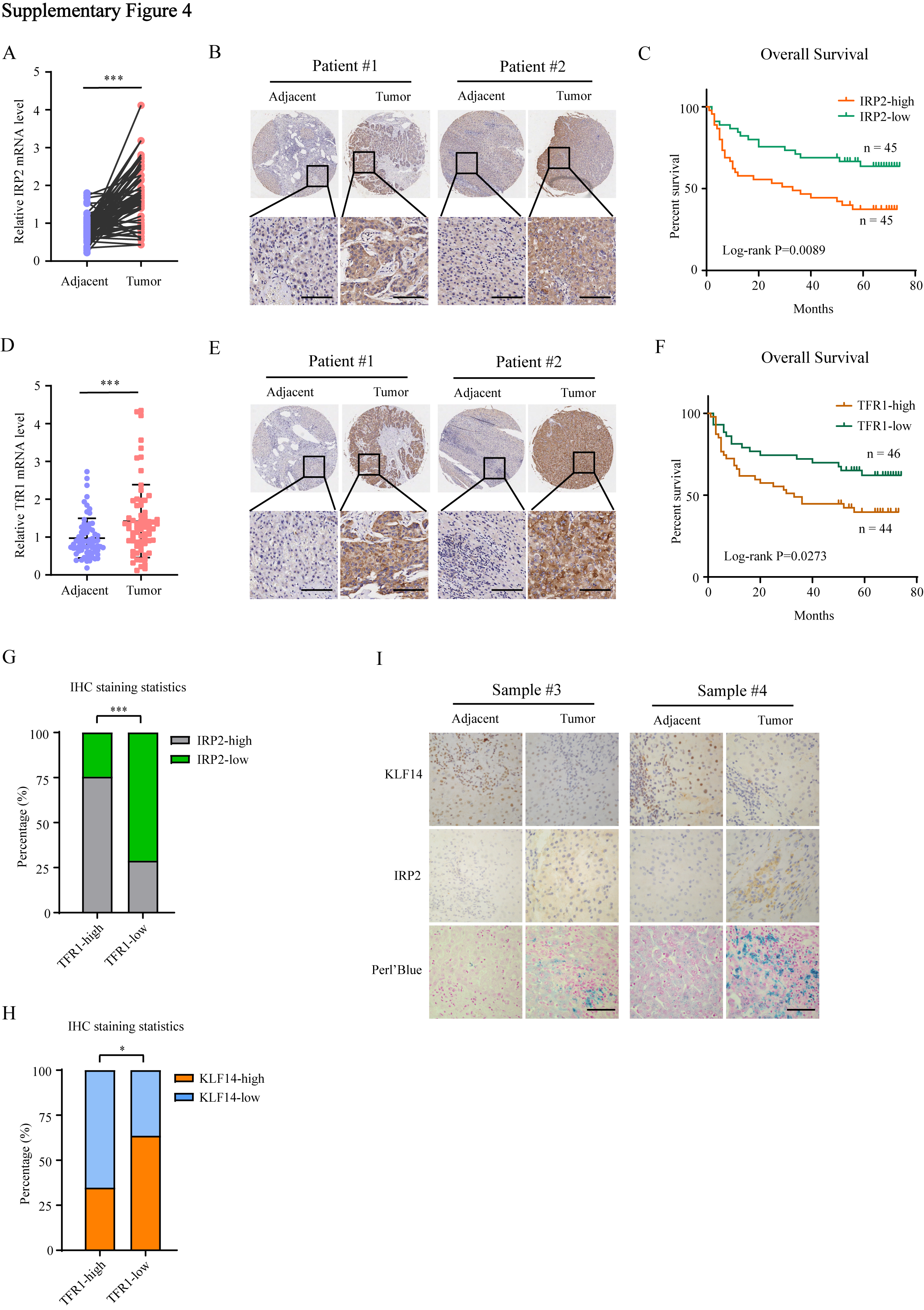

Supplement: Supplementary file 4 — Additional file 4: Supplementary Fig. 4. (A) Relative mRNA level of IRP2 in 69 paired tumor tissues and adjacent tissues of HCC. (B) Representative IHC staining of IRP2 in HCC tumor and adjacent tissues (n = 90) were exhibited (scale bar, 100 μm). (C) Kaplan-Meier analyses were conducted to evaluate the overall survival according to the expression of IRP2 in HCC tissue chip. (D) Relative mRNA level of TfR1 in 69 paired tumor tissues and adjacent tissues of HCC. (E) Representative IHC staining of TfR1 in HCC tumor and adjacent tissues were exhibited (scale bar, 100 μm). (F) Kaplan-Meier analyses were conducted to evaluate the overall survival according to the expression of TfR1 in HCC tissue chip. (G)(H) Statistics of IHC staining displayed the percentages of HCC specimens with higher or lower IRP2/KLF14 expression and corresponding TfR1 levels. (I) Immunohistochemistry staining of KLF14, IRP2 and Perl’s Blue (iron) in HCC tumor and adjacent tissues (n = 4) were exhibited (scale bar, 50 μm). Two-tailed unpaired Student’s T-tests were performed. *P < 0.05, and ***P < 0.001. [file 13046_2022_2562_MOESM4_ESM.tif]

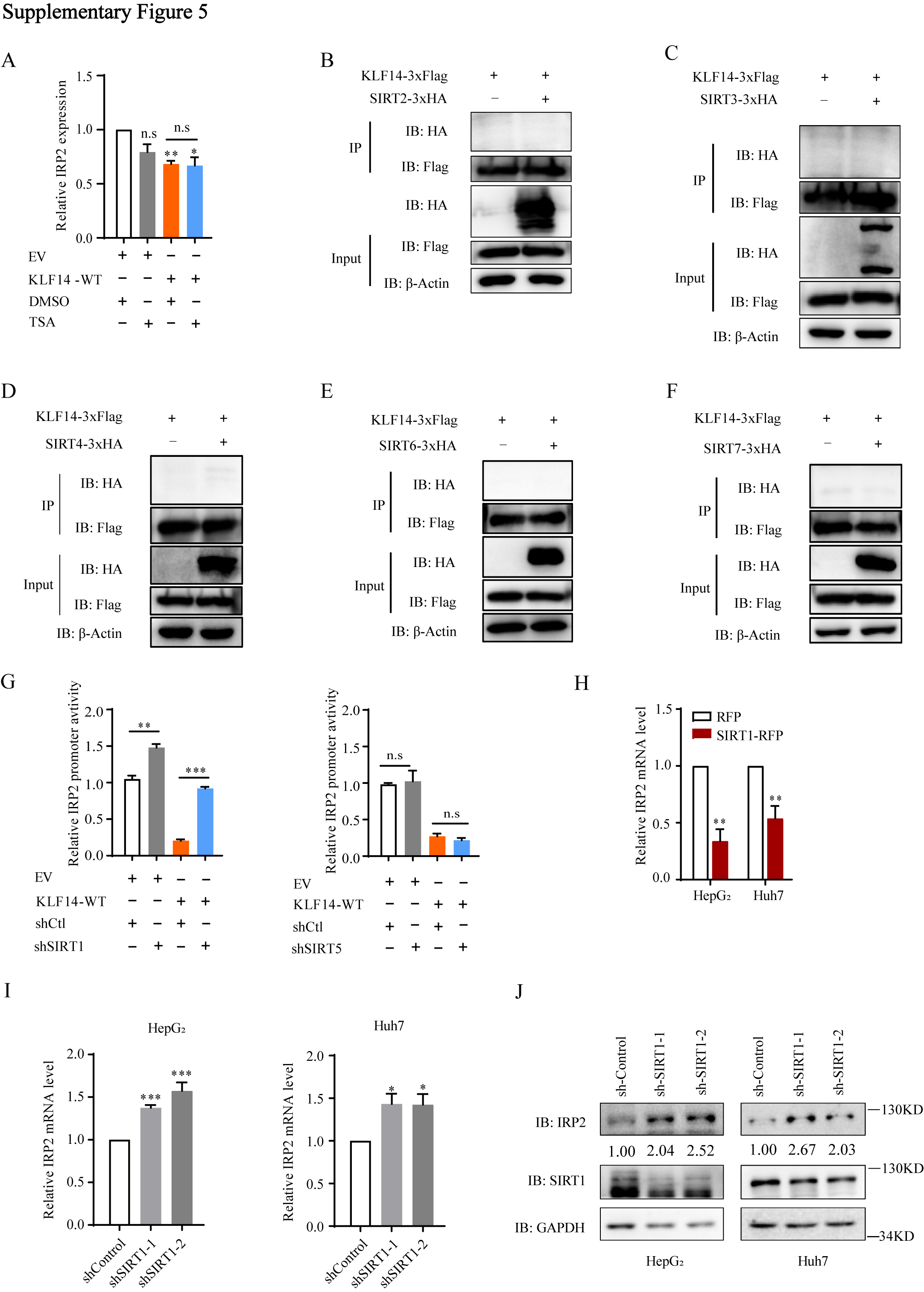

Supplement: Supplementary file 5 — Additional file 5: Supplementary Fig. 5. (A) Relative mRNA level of IRP2 in KLF14 overexpressed cells and/or TSA treatment. (B) SIRT2, 3, 4, 6, 7–3 × HA and KLF14-WT-3 × Flag were transfected into 293 T cells for 48 h, whole-cell lysates were immunoprecipitated with Flag beads and the co-precipitated HA was detected. (C) Silencing of SIRT1 but not SIRT5 reduced the suppression effects of KLF14 on IRP2 promoter activity. (E) Relative mRNA level of IRP2 in SIRT1 overexpressed cells. (F) Relative mRNA level of IRP2 in cells with SIRT1 silenced. (G) The protein level of IRP2 in cells with SIRT1 silenced. Data represent means ± SD, n.s, not significant, *P < 0.05, **p < 0.01, ***p < 0.001. [file 13046_2022_2562_MOESM5_ESM.tif]

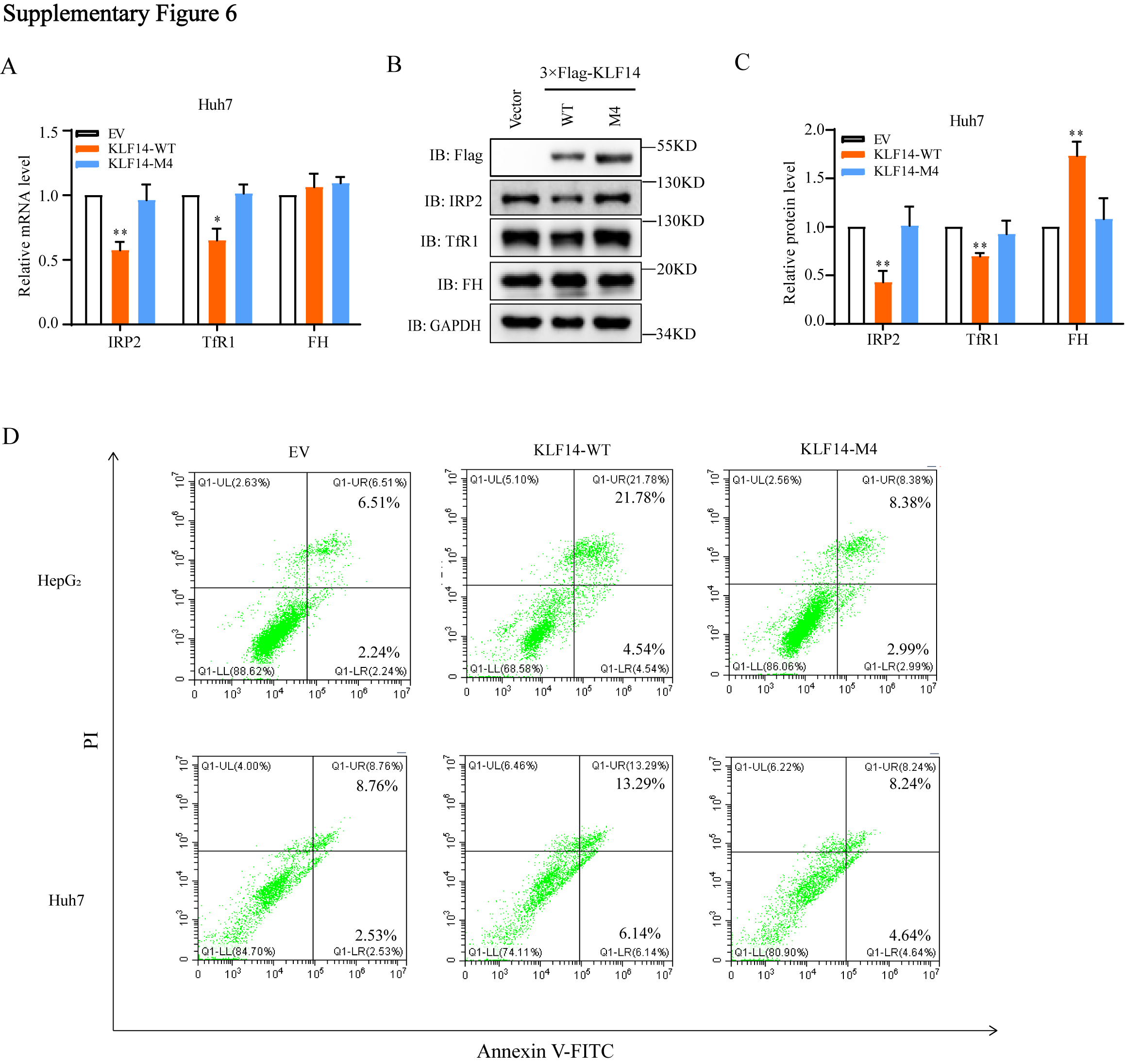

Supplement: Supplementary file 6 — Additional file 6: Supplementary Fig. 6. (A) Relative mRNA levels of IRP2, TfR1, FH and GAPDH (control) in KLF14-M4–3 × Flag overexpressed Huh7 cells. (B-C) The protein levels of IRP2, TfR1, FH and GAPDH (control) in KLF14-M4–3 × Flag overexpressed Huh7 cells. (D) Cell apoptosis of KLF14-M4–3 × Flag overexpressed cells were investigated by flow cytometry. Data represent means ± SD, n.s, not significant, *P < 0.05, **p < 0.01, ***p < 0.001. [file 13046_2022_2562_MOESM6_ESM.tif]

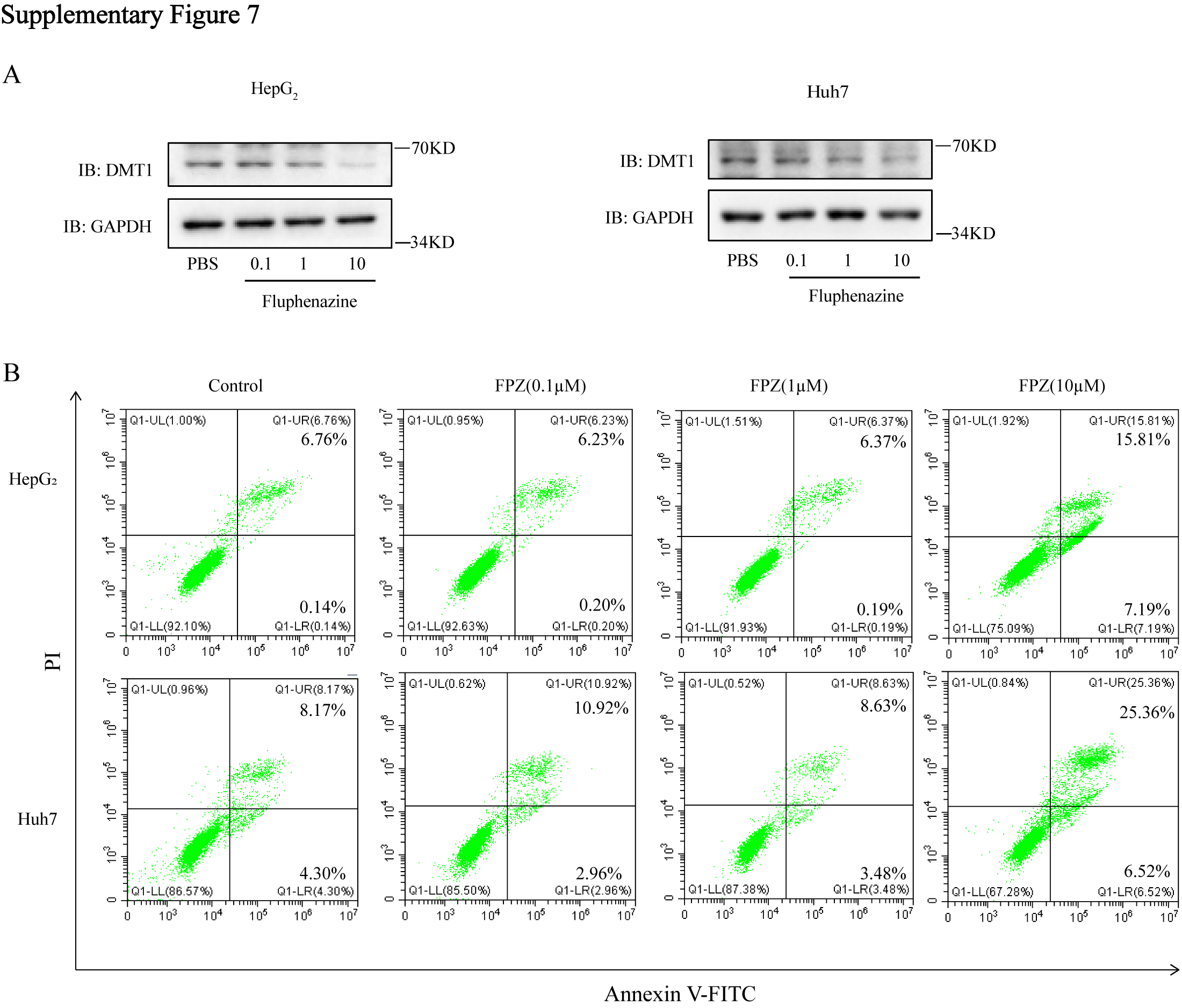

Supplement: Supplementary file 7 — Additional file 7: Supplementary Fig. 7. (A) The protein levels of DMT1 in HepG2 and Huh7 cells with fluphenazine treatment. (B) Cell apoptosis of HepG2 and Huh7 cells with incubation of PBS or fluphenazine at the indicated dosage for 48 h were investigated by flow cytometry. [file 13046_2022_2562_MOESM7_ESM.tif]

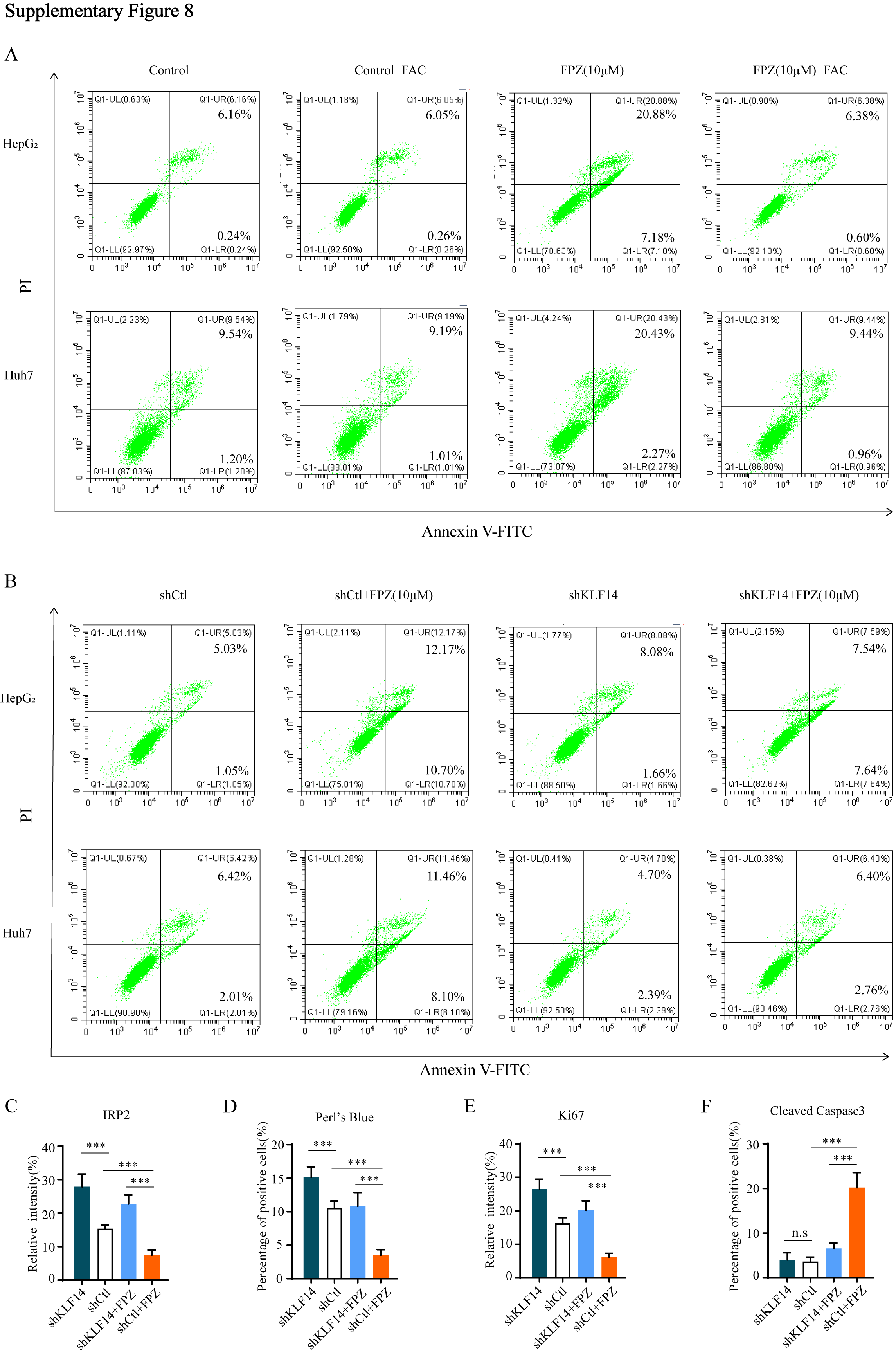

Supplement: Supplementary file 8 — Additional file 8: Supplementary Fig. 8. (A) Cell apoptosis of HepG2 and Huh7 cells with incubation of PBS, 10 μM Fluphenazine or the combination with 100 μM FAC for 48 h, were investigated by flow cytometry. (B) KLF14-silenced HepG2 and Huh7 cells were treated with PBS or 10 μM Fluphenazine for 48 h, then cell apoptosis was investigated by flow cytometry. (C-F) The relative intensities of IHC staining or percentage of positive cells in tumor tissues isolated from mice were quantified by Image J (version w.8.0). Data represent means ± SD, n.s, not significant, **p < 0.01, ***p < 0.001. [file 13046_2022_2562_MOESM8_ESM.tif]
