# Supplementary material for: KLF14 regulates the growth of hepatocellular carcinoma cells via its modulation of iron homeostasis through the repression of iron-responsive element-binding protein 2
Source: J Exp Clin Cancer Res. 2023 Jan 5;42:5. doi: 10.1186/s13046-022-02562-4 (PMC9814450; doi:10.1186/s13046-022-02562-4)
Supplement: Supplementary file 9 — Additional file 9: Table S1. Primers for qRT-PCR. Table S2. Primers for ChIP-qPCR. Table S3. Correlation between KLF14 expression and clinicopathologic features in patients with HCC. [file 13046_2022_2562_MOESM9_ESM.doc]

**Supplementary Table 1**

**Table S1. Primers for qRT-PCR**

| *GAPDH* | Forward | 5’-TCAGTGGTGGACCTGACCTG-3’ |
| --- | --- | --- |
|  | Reverse | 5’-TGCTGTAGCCAAATTCGTTG-3’ |
| *TFR2* | Forward | 5’-GACCCTGCAGTGGGTGTACT-3’ |
|  | Reverse | 5’-CAGTCGCTCGTCTCTCTCCT-3’ |
| *CP* | Forward | 5’-AGCCTTGAGGTTTCAGCCTGG-3’ |
|  | Reverse | 5’-AAGAACCTCAAACGTGGCCT-3’ |
| *TF* | Forward | 5’-TCAGCAGAGACCACCGAAGACT-3’ |
|  | Reverse | 5’-GACCACACTTGCCCGCTATGTA-3’ |
| *C19orf12* | Forward | 5’-GAAGGGTGCCCTGGTCACAGG-3’ |
|  | Reverse | 5’-GCAGCTCCATTAGGATCTGAGG-3’ |
| *ACO1(IRP1)* | Forward | 5’-TGCTTCCTCAGGTGATTGGCTACA-3’ |
|  | Reverse | 5’-TAGCTCGGTCAGCAATGGACAACT-3’ |
| *IRP2* | Forward | 5’-CGCCTTTGAGTACCTTATTGAAACA-3’ |
|  | Reverse | 5’-CGTACAGCAGCTTCCAACAAGA-3’ |
| *TfR1* | Forward | 5’-AGTTGAACAAAGTGGCACGAGCAG-3’ |
|  | Reverse | 5’-AGCAGTTGGCTGTTGTACCTCTCA-3’ |
| *FH* | Forward | 5’-ACTGATGAAGCTGCAGAACC-3’ |
|  | Reverse | 5’-GTCACCCAATTCTTTGATGG-3’ |
| *DMT1* | Forward | 5’-GTGTTCTACTTGGGTTGGCAATGT-3’ |
|  | Reverse | 5’-TTGGCTATGTTCACACAGTAAACCAT-3’ |
| *FPN* | Forward | 5’-GATCCTTGGCCGACTACCTG-3’ |
|  | Reverse | 5’-CACATCCGATCTCCCCAAGT-3’ |
| *KLF14* | Forward | 5’-TACAAGTCGTCGCACCTCAA-3’ |
|  | Reverse | 5’-GTCCCCGGTACTCGATCATA-3’ |
| *SIRT1* | Forward | 5’-TAGCCTTGTCAGATAAGGAAGGA-3’ |
|  | Reverse | 5’-ACAGCTTCACAGTCAACTTTGT-3’ |
| *SIRT5* | Forward | 5’-TTGGAGAAAACCTGGATCCTG-3’ |
|  | Reverse | 5’-CTGAATCTGTCGGTGGCTG-3’ |

**Supplementary Table 2**

**Table S2. Primers for ChIP-qPCR**

| *IRP2* Pro1  (TSS-966bp) | Forward | 5’- TTGGCCTCCTAAAGTGCTGG -3’ |
| --- | --- | --- |
|  | Reverse | 5’- CCATCAGCTTCTGTGACTTC -3’ |
| *IRP2* Pro2  (TSS-663bp) | Forward | 5’- GAGAGCCGAGTATGAGCGAC -3’ |
|  | Reverse | 5’- TTACCCTCCGGTAGCTTCCG -3’ |
| *IRP2* Pro3  (TSS-479bp) | Forward | 5’- TCATTCAACAGTTGAGCGCC -3’ |
|  | Reverse | 5’- ACTGCTAGGAGAAAGAGCTC -3’ |
| *IRP2* Pro4  (TSS-133bp) | Forward | 5’- GAAATCGCTTTCTGGTTAGC -3’ |
|  | Reverse | 5’- GGAAAGAAGGAAGCAGGCTC -3’ |
| TSS+219bp | Forward | 5’- GAGGGATAATATGGTCTCCG -3’ |
|  | Reverse | 5’- GCGAGAAAAGCAAGGAATTC -3’ |

**Supplementary Table 3**

**Table S3. Correlation between KLF14 expression and clinicopathologic features in patients with HCC**

| Characteristics |  |  | KLF14 Expression | | *p*-value |
| --- | --- | --- | --- | --- | --- |
|  |  | N | high | low |  |
| Age（year） | ≤50 | 40 | 20 | 20 | >0.9999 |
|  | ＞50 | 50 | 24 | 26 |  |
| Gender | Female | 20 | 7 | 13 | 0.2071 |
|  | Male | 70 | 37 | 33 |  |
| Tumor size (cm) | ≤5 | 54 | 35 | 19 | **0.0003^***^** |
|  | ＞5 | 36 | 9 | 27 |  |
| Tumor number | Single | 81 | 41 | 40 | 0.4856 |
|  | Mutiple | 9 | 3 | 6 |  |
| TNM stage | Ⅰ-Ⅱ | 74 | 44 | 30 | **<0.0001^***^** |
|  | Ⅲ-Ⅳ | 16 | 0 | 16 |  |
| PVTT | Absent | 65 | 38 | 27 | **0.0045^**^** |
|  | Present | 25 | 6 | 19 |  |
| Serum AFP level  (ng/mL) | ≤400 | 56 | 37 | 19 | **<0.0001^***^** |
|  | ＞400 | 34 | 7 | 27 |  |
| HBsAg | Negative | 19 | 10 | 9 | 0.7988 |
|  | Positive | 71 | 34 | 37 |  |

PVTT, portal vein tumor thrombus; AFP, alpha-fetoprotein; HBsAg, Hepatitis B surface Antigen.

The bold number means statistically significant. ***P* < 0.01 and ****P* < 0.001.
